# Nb_2_O_5_‐Based Photocatalysts

**DOI:** 10.1002/advs.202003156

**Published:** 2021-02-22

**Authors:** Kaiyi Su, Huifang Liu, Zhuyan Gao, Paolo Fornasiero, Feng Wang

**Affiliations:** ^1^ State Key Laboratory of Catalysis (SKLC) Dalian National Laboratory for Clean Energy (DNL) Dalian Institute of Chemical Physics (DICP) Chinese Academy of Sciences Dalian 116023 China; ^2^ University of Chinese Academy of Sciences Beijing 100049 China; ^3^ Department of Chemical and Pharmaceutical Sciences INSTM ‐ Trieste and ICCOM ‐ CNR Trieste University of Trieste Via L. Giorgieri 1 Trieste 34127 Italy

**Keywords:** acidity, Nb_2_O_5_, photocatalysis, photocatalysts, photodegradation, photooxidation

## Abstract

Photocatalysis is one potential solution to the energy and environmental crisis and greatly relies on the development of the catalysts. Niobium pentoxide (Nb_2_O_5_), a typically nontoxic metal oxide, is eco‐friendly and exhibits strong oxidation ability, and has attracted considerable attention from researchers. Furthermore, unique Lewis acid sites (LASs) and Brønsted acid sites (BASs) are observed on Nb_2_O_5_ prepared by different methods. Herein, the recent advances in the synthesis and application of Nb_2_O_5_‐based photocatalysts, including the pure Nb_2_O_5_, doped Nb_2_O_5_, metal species supported on Nb_2_O_5_, and other composited Nb_2_O_5_ catalysts, are summarized. An overview is provided for the role of size and crystalline phase, unsaturated Nb sites and oxygen vacancies, LASs and BASs, dopants and surface metal species, and heterojunction structure on the Nb_2_O_5_‐based catalysts in photocatalysis. Finally, the challenges are also presented, which are possibly overcome by integrating the synthetic methodology, developing novel photoelectric characterization techniques, and a profound understanding of the local structure of Nb_2_O_5_.

## Graphical abstract

Niobium pentoxide (Nb_2_O_5_), a typically nontoxic metal oxide, exhibits unique redox ability and acidity. Recently, the applications of Nb_2_O_5_‐based photocatalysts are reported in the photodegradation of plastics, reduction of CO_2_, photocatalytic hydrogen evolution, and organic synthesis. Herein, an overview is provided for the synthesis, application of Nb_2_O_5_‐based photocatalysts, and corresponding structure–activity relationship.

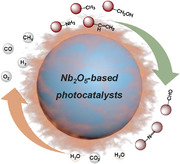

## 1 Introduction

Abundant fossil resources are utilized to fulfill the growing energy and chemical requirements.^[^ ^1^ ^]^ However, carbon dioxide generated in these processes is inevitably released into the environment, accompanying global warming, ocean acidification, and a series of ecological problems.^[^ ^2^ ^]^ Moreover, the expected global energy consumption is up to 22.5 trillion watts (22.5 TW) of power demand in 2030.^[^ ^3^ ^]^ Notably, electromagnetic radiation power flow from the Sun on the Earth is estimated to be 120 000 TW, which is far beyond the global energy consumption without carbon emission.^[^ ^3^ ^]^ Learning from photosynthesis in nature, photocatalysis is potentially utilized for scalable and controlled production of fuel and diverse chemicals to alleviate the dependence on fossil fuels and the consequent environmental pollution.^[^ ^4^ ^]^ Nowadays, diverse semiconductors are synthesized and applied in the photocatalytic process.^[^ ^5^ ^]^ In principle, the electrons are motivated by the light and then transfer from valence band (VB) to conduction band (CB) in semiconductor photocatalysts, which induce subsequent redox reactions.^[^ ^6^ ^]^ For instance, metal oxide, metal sulfide, metal nitride, metal phosphide, and nonmetallic material, like carbon nitride, are reported in the areas of photocatalysis, such as pollutant degradation, hydrogen generation, chemical synthesis, etc.^[^ ^7^ ^]^ In these studies, researchers are devoted to preparing the photocatalysts that are nontoxic, eco‐friendly, low‐cost, and efficient, to realize reactions under mild conditions with massive desired products.

**Figure 1:**
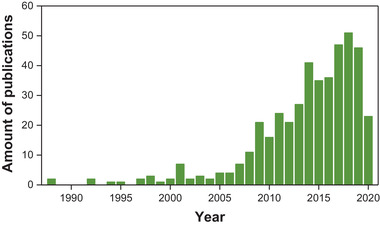
The amount of publications from the Web of Science by searching the keywords“Nb2O5” and “photocatal*” on May 30, 2020.

The niobium pentoxide (Nb_2_O_5_), a typically nontoxic solid oxide, exhibits strong redox ability and unique Lewis acid sites (LASs) and Brønsted acid sites (BASs).^[^ ^4^, ^8^ ^]^ Previously, Ziolek's group and Tsang's group mentioned the photocatalytic performance of different niobium compounds and nanostructured Nb_2_O_5_ in 1999 and 2012, respectively.^[^ ^4^, ^8^ ^]^ Recently, the amount of publications in Nb_2_O_5_ photocatalyst increased rapidly over the past decade (**Figure** **1**), indicating the novel discovery and profound understanding of Nb_2_O_5_. Concretely, the applications of Nb_2_O_5_ are extended to the photocatalytic conversion of waste plastics, activation of hydrocarbon, photoreduction of CO_2_, and selective transformation of amines and alcohols.^[^ ^9^ ^]^ For instance, Nb_2_O_5_ exhibited a higher reaction rate and selectivity than those of TiO_2_ in the selective photooxidation of benzylamine to *N*‐benzylidene benzylamine.^[^ ^9c^ ^]^ Besides, inert polyethylene and waste plastics were completely degraded on Nb_2_O_5_ at 25 °C, while the generated CO_2_ was further reduced to CH_3_COOH.^[^ ^9a^ ^]^ The selectivity of CO_2_ to CO, CH_4_, and other acid products is related to the distribution of LAS and BAS on the Nb_2_O_5_ surface.^[^ ^9b^ ^]^ These results suggest the attractive properties of Nb_2_O_5_ and its potential in practical applications. However, a few review articles systematically summarize Nb_2_O_5_‐based photocatalysts to provide the structure–activity relationship for future studies.

**Figure 2:**
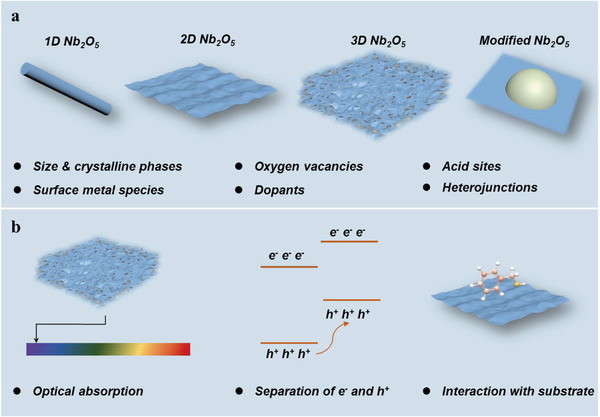
An overview of a) Nb_2_O_5_‐based photocatalysts and b) plausible influence in photocatalysis.

Hence, we provide an overview of the recent advances in different Nb_2_O_5_‐based photocatalysts, including the synthesis, application, and relationship between the photoelectronic properties, surface structures, and activities. First, the physicochemical properties of Nb_2_O_5_ are introduced. Then, reported Nb_2_O_5_‐based photocatalysts are classified into two categories: i) pure Nb_2_O_5_ catalysts with diverse morphology, and ii) Nb_2_O_5_ catalysts with other species, such as metal species and other components. In the synthetic sections, we summarize the method and the role of treatment and additives in the control of morphology and structure. After that, we discuss the optical and catalytic properties of Nb_2_O_5_‐based photocatalysts (**Figure** **2**). Except for the generation, migration, and recombination of charge carriers, acidic properties and Nb—O—metal interface are also taken into account, which can affect the interaction between substrate molecules and catalyst, the product selectivity, and reaction rate. In the end, we give the summary and outlook of Nb_2_O_5_‐based photocatalysts.

## 2 The Nature of Nb_2_O_5_

### 2.1 Resource

The abundance of niobium on earth is 20 ppm.^[^ ^8a^ ^]^ Niobium does not exist in a pure metallic form but is often found as mixtures with other metals in minerals, which are unevenly distributed around the globe.^[^ ^10^ ^]^ Abundant columbic mines are mainly located in Brazil, Canada, and Nigeria.^[^ ^8a^ ^]^ Nowadays, niobium compounds are widely utilized in superconducting, electronics, and catalytic industries, indicating that the recovery of niobium‐containing solid waste is potential strategies to produce desired niobium oxide.^[^ ^11^ ^]^ In the field of catalysis, niobium powders and Nb_2_O_5_ obtained from the minerals are the raw material for the production of niobium chloride, niobium oxalate, ammonia niobium oxalate, niobium pentabutoxide, and other organic niobium salts, which can be utilized for further preparation of nanostructured Nb_2_O_5_ due to the differences in acidity, alkalinity, and solubility.^[^ ^4^, ^8^, ^12^ ^]^

### 2.2 Physicochemical Properties

**Figure 3:**
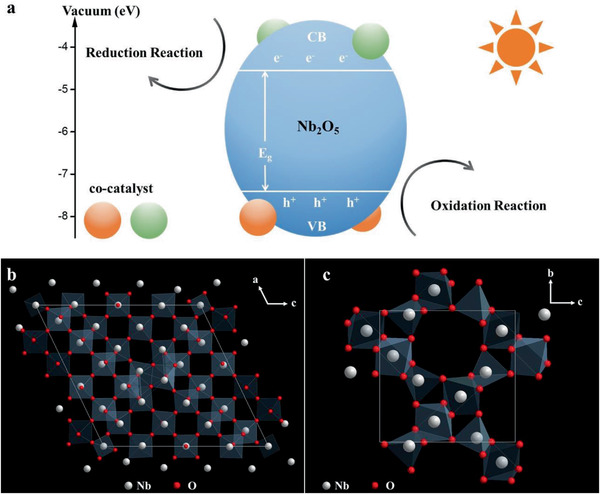
a) The photocatalytic process on Nb_2_O_5_, and the local structure of b) H‐Nb_2_O_5_ and c) T‐Nb_2_O_5_.^[^ ^8^, ^19^ ^]^ a) Adapted with permission.^[^ ^19^ ^]^ Copyright 2014, Royal Society of Chemistry. b,c) Adapted with permission.^[^ ^8a^ ^]^ Copyright 1999, American Chemical Society.

The Nb_2_O_5_ is an n‐type semiconductor.^[^ ^13^ ^]^ The structure of Nb_2_O_5_ depends on the preparation conditions.^[^ ^14^ ^]^ Amorphous Nb_2_O_5_ can be transformed into pseudohexagonal phase (TT‐Nb_2_O_5_), orthorhombic phase (T‐Nb_2_O_5_), and monoclinic phase (H‐Nb_2_O_5_) by increasing the temperature.^[^ ^8a^ ^]^ Unique properties are observed in Nb_2_O_5_. Nb_2_O_5_ has the bandgap energy (*E*_g_) value of ≈3.0–3.4 eV, which is suitable for redox reaction in photocatalysis.^[^ ^4a^ ^]^ As shown in **Figure** **3**a, the excited electrons and holes on Nb_2_O_5_ migrate to the surface under light irradiation and then interact with the substrate in the reduction process and oxidation process, respectively. Besides, LASs and BASs are observed on tetrahedral NbO_4_ and octahedral NbO_6_ units (Figure 3b,c), respectively.^[^ ^14^, ^15^ ^]^ The Nb_2_O_5_ exhibits high acid strength and is utilized in the dehydration reaction, hydrolysis reaction, and hydrodeoxygenation reaction.^[^ ^8^, ^16^ ^]^ Meanwhile, these structures can be distorted by decreasing the thickness of Nb_2_O_5_ and reduction with the formation of Nb_2_O_5−_ *_x_* and NbO_2_.^[^ ^15^, ^17^ ^]^ In addition, H_2_O and hydroxyl groups on the Nb_2_O_5_ surface can be removed by post‐treatment, resulting in the improvement of absorption performance.^[^ ^18^ ^]^ Except for the unique acidity and redox properties, Nb_2_O_5_ is robust in organic acid solutions. This property implies that Nb_2_O_5_ can be stable in acidic solutions derived from biomass and other acid products in photocatalysis. However, there are still some limitations of Nb_2_O_5_. An unavoidable trade‐off is present between the restricted optical absorption and suitable photoredox ability of Nb_2_O_5_. In addition, the methods are still necessary for the large‐scale production of specific morphology of Nb_2_O_5_‐based photocatalysts. Thus, an overview is conducive to the systematic understanding and development of Nb_2_O_5_‐based photocatalysts.

## 3 Synthesis of Nb_2_O_5_‐Based Photocatalysts

### 3.1 Synthesis of Pure Nb_2_O_5_ Catalysts

The specific surface area (SSA) of commercial Nb_2_O_5_ (orthorhombic phase) is lower than 15 m^2^ g^−1^.^[^ ^17b^ ^]^ This property leads to the restricted active sites and a high recombination efficiency of charge carriers on Nb_2_O_5_.^[^ ^20^ ^]^ Nowadays, the Nb_2_O_5_ catalysts with diverse morphologies are synthesized to solve these problems in photocatalysis.^[^ ^8e^ ^]^ These catalysts can be divided into three classes: 1D, 2D, and 3D Nb_2_O_5_ catalysts. Although 0D Nb_2_O_5_ quantum dots (QDs) were synthesized by the physical vapor deposition, the application of Nb_2_O_5_ QDs in photocatalysis is rarely reported.^[^ ^21^ ^]^ Therefore, the synthesis and applications of Nb_2_O_5_ QDs are not mentioned in this work.

#### 3.1.1 1D Nb_2_O_5_ Catalysts

Recently, diverse methods have been developed for the synthesis of 1D Nb_2_O_5_ nanorods, nanowires, and nanotubes. In 2006, the preparation of Nb_2_O_5_ nanotubes was reported by the atomic layer deposition (ALD) approach.^[^ ^22^ ^]^ Amorphous Nb_2_O_5_ was deposited first on the porous Al_2_O_3_ template with gas pulses of niobium iodide (NbI_5_) and oxygen.^[^ ^22^ ^]^ Then, Al_2_O_3_ was removed by chromic acid/phosphoric acid solution to produce desired Nb_2_O_5_ nanotubes.^[^ ^22^ ^]^ Because of the costly apparatus and low yield of catalysts in the ALD process, other synthesis methods are necessary. The 1D T‐Nb_2_O_5_ and TT‐Nb_2_O_5_ nanotubes were obtained from layered niobates.^[^ ^23^ ^]^ First, layered K_4_Nb_6_O_17_ was synthesized from the solid reaction of Nb_2_O_5_ and K_2_CO_3_ under the calcination. Then, the scrolled H_4_Nb_6_O_17_ was prepared via the exfoliation of K_4_Nb_6_O_17_ with the assistance of acid and base. Finally, the Nb_2_O_5_ nanotubes were obtained from H_4_Nb_6_O_17_ by dehydration under 400–450 °C. The heat treatment was a vital process for the transformation of nanosheets to nanotubes.^[^ ^23^ ^]^ Besides, the Kirkendall effect was applied in the synthesis of H‐Nb_2_O_5_ nanotubes from nanorods by a two‐step hydrothermal synthesis approach.^[^ ^24^ ^]^ The TT‐Nb_2_O_5_ nanorod arrays grew on niobium foils in the first hydrothermal process. Due to the Kirkendall effect, the outside walls of TT‐Nb_2_O_5_ nanorods exhibited preferential nucleation and growth in the second hydrothermal process, which is accompanied by the migration of inside core composition and the formation of nanotubes. After that, a one‐step hydrothermal method was reported.^[^ ^25^ ^]^ The Nb_2_O_5_ powders, hydrofluoric acid (HF), hydrogen peroxide, and Ti powders were introduced into the precursor. Hydrofluoric acid acted as an etching reagent to disperse Nb powders in solution.^[^ ^25^, ^26^ ^]^ In addition, the evolution of nanotubes was significantly affected by the concentration of F^−^ ions. This phenomenon may be due to the fact that F^−^ anions act as a structure‐directing agent to control the crystal growth.^[^ ^25^, ^26^ ^]^ Similarly, T‐Nb_2_O_5_ nanotubes were obtained by the electrochemical method, which consisted of the anodization of Nb with ammonium fluoride.^[^ ^27^ ^]^ However, toxic reagents (e.g., HF, NH_4_F, or H_2_O_2_) are required in these processes.

Compared to the nanotubes, Nb_2_O_5_ nanorods can be synthesized directly from Nb probes and foils by a calcination method at ≈1000 °C.^[^ ^28^ ^]^ Besides, the decomposition of niobium isopropoxide was controlled to prepare the Nb_2_O_5_ nanorods in chemical vapor deposition (CVD) at 950 °C.^[^ ^12d^ ^]^ The synthesis methods of Nb_2_O_5_ nanorods were reported at lower temperatures in other studies. For instance, TT‐Nb_2_O_5_ nanorods encased in carbon were obtained from the niobium ethoxide by calcination at 800 °C in the 3 mL autoclave under nitrogen.^[^ ^29^ ^]^ To remove the carbonaceous residues, the as‐synthetic material was further treated at 500 °C under air condition, leading to the formation of T‐Nb_2_O_5_ nanorods.^[^ ^29^ ^]^ Besides, the topochemical method was developed, which is composed of the i) synthesis of specific morphology of niobates, ii) ion‐exchange for removal of other metal ions on niobates, and iii) calcination for the phase transformation.^[^ ^30^ ^]^ Typically, the KNb_3_O_8_ nanowires were prepared by molten salts of Nb_2_O_5_ and KCl under 800 °C and treated with HNO_3_ to produce H_3_ONb_3_O_8_ nanorods, which were further calcinated to produce the H‐Nb_2_O_5_ nanorods.^[^ ^30b^ ^]^ Similarly, CaNb_2_O_6_ nanowires were also utilized to prepare the H_2_Nb_2_O_6_ nanorods, which were further transformed to produce the T‐Nb_2_O_5_ nanorods.^[^ ^31^ ^]^ Additionally, the solvothermal approach was reported in the catalyst preparation, following the calcination treatment to prepare the Nb_2_O_5_ nanorods from amorphous Nb_2_O_5_·*n*H_2_O. The additives, like alcohol, played a key role in the hydrothermal process.^[^ ^32^ ^]^ If alcohols are present in the solution, the Nb_2_O_5_ particles were observed. According to this phenomenon, TT‐Nb_2_O_5_ nanorods were synthesized by a one‐step alcohothermal method, which is contributed to the growth direction of [001] for the 1D structure under enough reaction time, temperature, and concentration of NbCl_5_.^[^ ^32^ ^]^ Likewise, TT‐Nb_2_O_5_ nanorods were obtained by introducing oleic acid and trioctylamine, isopropanol, benzyl alcohol (BA), and triethylamine in the hydrothermal process.^[^ ^12^, ^33^ ^]^ In addition, corrosive NH_4_F and H_2_O_2_, HF, cetyltrimethylammonium bromide, and ionic liquid are also effective additives.^[^ ^34^ ^]^ Particularly, the rodlike structure was observed when the hydrothermal process was prolonged to 30 days without any additives.^[^ ^35^ ^]^ Electrospinning was also utilized in the synthesis of Nb_2_O_5_ nanorods.^[^ ^36^ ^]^ A mixture of Nb(OEt)_5_, polyvinylpyrrolidone (PVP), acetic acid, and ethanol solution was prepared before the electrospinning. The complex of PVP and acetic acid in the solution acted as a template.^[^ ^36^ ^]^ After electrospinning operation, the obtained material was treated at 550 °C to produce the Nb_2_O_5_ nanorods under air condition.^[^ ^36^ ^]^ In these methods, calcination is generally utilized for the removal of carbonaceous impurities or the change in the crystallinity.

For the synthesis of Nb_2_O_5_ nanowires, a thermal oxidation approach was reported from the linear Nb foils under 900–1000 °C.^[^ ^37^ ^]^ Similarly, Nb_2_O_5_ nanowires can be obtained from the topochemical method. T‐Nb_2_O_5_ was synthesized from NaNbO_3_ nanowires under 700 °C.^[^ ^38^ ^]^ H‐Nb_2_O_5_ nanowires were also prepared from Nb_3_O_7_(OH) nanorods by calcination at 450 °C.^[^ ^39^ ^]^ Besides, TT‐Nb_2_O_5_ nanowires can be prepared with the assistance of reflux.^[^ ^40^ ^]^ The refluxing is a useful approach to synthesize metal oxide nanorods with suitable additives for crystal growth, like trioctylamine, which directly affects the pH, the hydrolysis, and deposition of the precursor.^[^ ^40^ ^]^ When the precursor solution tends to be acidic, high crystallinity of Nb_2_O_5_ nanowires is observed after calcination.^[^ ^40^ ^]^ In addition, electrospinning was also reported in the preparation of T‐Nb_2_O_5_ nanowires.^[^ ^41^ ^]^ Furthermore, Nb foil was treated with oxygen plasma to induce the growth of Nb_2_O_5_ nanowires over the Nb foil.^[^ ^42^ ^]^

#### 3.1.2 2D Nb_2_O_5_ Catalysts

Nb_2_O_5_ nanosheets are typical 2D materials, which are attractive due to their unique structure and electronic properties. Previously, Nb_2_O_5_ nanosheets could be fabricated directly from the raw Nb_2_O_5_ and NbCl_5_ without any templates or organic polymers.^[^ ^43^ ^]^ For instance, ≈3–5 nm T‐Nb_2_O_5_ nanosheets were produced from commercial NbO_2_ particles in a solution containing ethanol and urea under 130 °C for 30 days.^[^ ^44^ ^]^ Similarly, TT‐Nb_2_O_5_ nanosheets were also synthesized from NbCl_5_ in ethylenediamine solution by hydrothermal treatment and calcination.^[^ ^45^ ^]^ In this process, the alkaline additive may be beneficial for the nanosheet evolution.^[^ ^46^ ^]^ Especially, Wang's group reported the synthesis of the Nb_2_O_5_·*x*H_2_O nanosheets from NbCl_5_ by a one‐step hydrothermal method and revealed the effect of additives.^[^ ^47^ ^]^ They speculated that the alkaline NH_3_·H_2_O may play a key role in nanosheet synthesis. To prove the opinion, they replaced the NH_3_·H_2_O with other alkaline additives, like the NaOH, *n*‐butyl amine, and *t*‐butylammonium hydroxide (TBAOH).^[^ ^47^ ^]^ Nanosheetlike morphology is only obtained using *n*‐butyl amine and TBAOH, suggesting the vital role of the NH_4_^+^ ions. The ions in the Nb_2_O_5_ nanosheets, like NbO_4_^3−^, NbO_5_^5−^, and NbO_6_^7−^, exhibit negative charges, which show an electrostatic interaction with NH_4_^+^ ions that act as a capping agent. This interaction can restrain the interlamination growth and avoid the formation of bulk Nb_2_O_5_.^[^ ^47^ ^]^

**Figure 4:**
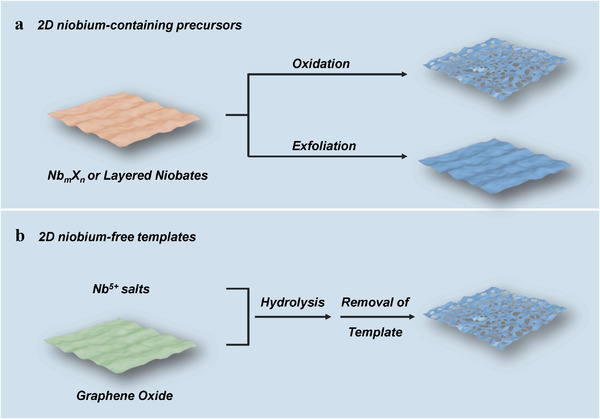
Synthesis of Nb_2_O_5_ nanosheets with the assistance of 2D a) niobium‐containing precursors and b) niobium‐free templates.

In addition to NbO_2_ and NbCl_5_, other 2D columbic compounds are also applied in the synthesis of Nb_2_O_5_ nanosheets by the topochemical method (**Figure** **4**a). For instance, 2D Nb*_m_*X*_n_* (X = Se and S) materials were utilized to prepare the Nb_2_O_5_ nanosheets.^[^ ^48^ ^]^ The Nb^4+^ species in NbSe_2_ oxidized to Nb^5+^ ions with the formation of Se under calcination, leading to the generation of porous Nb_2_O_5_ nanosheet.^[^ ^48^ ^]^Besides, the Nb_3_O_7_F nanosheets were produced by a hydrothermal approach and further calcinated to prepare the T‐Nb_2_O_5_ nanosheets.^[^ ^49^ ^]^ Additionally, other niobates were also reported in the synthesis of Nb_2_O_5_ nanosheets.^[^ ^17^, ^50^ ^]^ For instance, layered KNb_3_O_8_ was prepared by calcination of commercial Nb_2_O_5_ and K_2_CO_3_.^[^ ^17c^ ^]^ After that, the layered HNb_3_O_8_ was obtained from the KNb_3_O_8_ in an acidic solution by ion‐exchange.^[^ ^17c^ ^]^ Ultimately, few‐layer HNb_3_O_8_ nanosheets were observed by the intercalation of TBAOH.^[^ ^17c^ ^]^ Similarly, T‐Nb_2_O_5_ nanosheets were obtained from the layered H_4_Nb_6_O_17_·3H_2_O.^[^ ^51^ ^]^

Besides, layered templates are applied in the synthesis of Nb_2_O_5_ nanosheets (Figure 4b). The graphene oxide (GO), a typical 2D material, was introduced into the cyclohexane solution, following the addition of Nb(OC_4_H_9_)_5_.^[^ ^52^ ^]^ After the hydrothermal process, NbO*_x_*/GO nanosheets were observed.^[^ ^52^ ^]^ Subsequently, ≈2–4 nm Nb_2_O_5_ nanosheets were obtained by the removal of GO under calcination.^[^ ^52^ ^]^ Moreover, when the calcination temperature was elevated to 750 °C, holey T‐Nb_2_O_5_ nanosheets were observed by the utilization of GO.^[^ ^53^ ^]^ Likewise, silica/graphene nanosheets were utilized to prepare T‐Nb_2_O_5_ nanosheets by the hydrolysis of NbCl_5_.^[^ ^54^ ^]^ The silica in the template was removed by NaOH, maintaining the porous structure of Nb_2_O_5_.^[^ ^54^ ^]^ These preparation methods of Nb_2_O_5_ nanosheets can be divided into two classes. One kind of method is to synthesize layered intermediate that contains niobium to prepare nanosheets. Another one is to afford 2D templates that are niobium‐free to induce the growth of Nb_2_O_5_ nanosheets.

Furthermore, 2D ordered porous Nb_2_O_5_ were prepared with the assistance of chain ligand.^[^ ^55^ ^]^ In 1996, Ying's group put forward a method to synthesize the mesoporous Nb_2_O_5_ molecular sieve with the assistance of ligands.^[^ ^55^ ^]^ In this approach, Nb(OEt)_5_ and tetradecylamine were utilized to produce ringed Nb_2_O_5_ via hydrolysis, which is controlled precisely by the volume of water, hydrolysis temperature, and time.^[^ ^55^ ^]^ Because of the interaction between the basic amine ligand and Nb_2_O_5_, the residual tetradecylamine molecules were further removed by strong acid HNO_3_/EtOH.^[^ ^55^ ^]^ After that, block‐copolymer/inorganic‐salt methodology was developed.^[^ ^56^ ^]^ The tetradecylamine can be replaced by poly (alkylene oxide) block copolymer, such as P‐123.^[^ ^56^ ^]^ Meanwhile, inorganic niobium salts, NbCl_5_, were also utilized as a precursor in an ethanol solution.^[^ ^56^ ^]^ The ordered mesoporous Nb_2_O_5_ was obtained by the formation of crown‐ether‐type complexes between alkylene oxide segments and inorganic ions through weak coordination bonds.^[^ ^56^ ^]^ Especially, when inorganic Nb salts and aqueous solution were introduced simultaneously into the precursor with controlled hydrolysis, 3D mesoporous Nb_2_O_5_ were observed.^[^ ^57^ ^]^ In addition to the utilization of a single hydrophilic ligand, amphiphilic block copolymers were also developed to fabricate Nb_2_O_5_ materials. The poly(ethylene‐*co*‐butylene)‐*b*‐poly(ethylene oxide) diblock copolymers were mixed with NbCl_5_ in the ethanol solution, following the calcination to remove the polymers and obtain the 2D mesoporous Nb_2_O_5_.^[^ ^58^ ^]^ The orientation of porous structure can be regulated by the changes in polymer hydrophilicity and hydrophobicity.^[^ ^59^ ^]^ For instance, the polymers with different chain lengths, like amphiphilic L64, P85, and P103, were effective in the synthesis of porous Nb_2_O_5_.^[^ ^59^ ^]^

#### 3.1.3 3D Nb_2_O_5_ Catalysts

Generally, the porous structure is beneficial for the diffusion and transmission of substrates.^[^ ^60^ ^]^ Although the porous Nb_2_O_5_, HY‐340, is supplied from the CBMM (Brazil, one commercial company), many research groups are still devoted to designing and developing novel synthetic methods of 3D porous Nb_2_O_5_ catalysts.

To date, 3D porous Nb_2_O_5_ catalysts can be synthesized from the Nb foils, niobium salts, and raw Nb_2_O_5_. The Nb foils were irradiated within a constant flux of 100 eV He^+^ ions under 500–950 °C to prepare the porous Nb_2_O_5_.^[^ ^61^ ^]^ With the increase of temperature, the pore diameter over Nb_2_O_5_ was larger, which can be up to 230 nm.^[^ ^61^ ^]^ In addition, Nb(OH)_5_ was obtained by the anodization of Nb foil in ethylene glycol containing 4 vol% HF and 2 vol% H_2_O_2_. Then, mesoporous Nb_2_O_5_ was obtained by the calcination of Nb(OH)_5_.^[^ ^62^ ^]^ In the anodization process, the porous structure was controlled by the changes in voltage, electrolyte temperature, time, and solution.^[^ ^17^, ^62^, ^63^ ^]^ Particularly, a careful cleaning process is necessary to remove the impurity on the surface of Nb foils before anodization.^[^ ^63d^ ^]^ Besides, the sol–gel approach was also reported. Nb(OC_2_H_5_)_5_ was hydrolyzed with the assistance of the NH_3_·H_2_O solution and calcined at 300 and 650 °C to TT‐Nb_2_O_5_ and T‐Nb_2_O_5_, respectively.^[^ ^64^ ^]^ Similarly, wormhole‐like amorphous Nb_2_O_5_ and hierarchically porous Nb_2_O_5_ were prepared from the hydrolysis of Nb salts (NbCl_5_ and Nb(OC_2_H_5_)_5_) by the addition of P‐123 and surfactant (Brij 56), respectively.^[^ ^20^, ^65^ ^]^ Other uniform templates, like polystyrene spheres and zeolites, were also utilized to prepare the porous Nb_2_O_5_. For instance, polystyrene spheres were introduced into the Nb–citric complex solution via the reaction of Nb_2_O_5_ with HF, NH_3_·H_2_O, and citric acid.^[^ ^66^ ^]^ The template was subsequently removed by the calcination with the formation of macroporous Nb_2_O_5_.^[^ ^66^ ^]^ The macropores size of Nb_2_O_5_ was greatly dependent on the diameter of the polystyrene spheres. Similarly, the ammonium niobate oxalate was deposited on the FDU‐1, one type of zeolite, by an impregnation method.^[^ ^67^ ^]^ Then, the FDU‐1 is removed by a diluted NaOH solution. Furthermore, T‐Nb_2_O_5_ and TT‐Nb_2_O_5_ were obtained with the assistance of other porous carbon materials, such as cotton.^[^ ^68^ ^]^ In these methods, the evolution of ordered porous structure mainly depended on the hydrolysis of niobium salt and the uniformity of templates.^[^ ^57^ ^]^

Porous Nb_2_O_5_ constituted by stacked particles was also reported.^[^ ^69^ ^]^ For instance, T‐Nb_2_O_5_ can be prepared by direct calcination of Nb powders.^[^ ^69^ ^]^ In addition, the hydrolysis of organic Nb salts was also applied in the synthesis of nanoparticles. Especially, Nb(OBu)_5_ was dissolved in toluene with different amounts of water in an autoclave under 300 °C for 2 h.^[^ ^70^ ^]^ When the amount of water was up to 30 cm^3^, amorphous Nb_2_O_5_ were transformed to TT‐phase and grew from ≈5 to 30–60 nm, indicating that water was beneficial for the dissolution–recrystallization process on the growth of Nb–O–Nb structure.^[^ ^70^ ^]^ Instead of toluene, ethanol, triethylamine, and H_2_O_2_ solution were also applied in the synthesis of Nb_2_O_5_ particles.^[^ ^71^ ^]^ Besides, the NbCl_5_ and Nb‐fluoro complex were used in the synthesis of H‐Nb_2_O_5_ and TT‐Nb_2_O_5_ particles, respectively.^[^ ^72^ ^]^ In the hydrolysis process, structure‐directing agents were introduced, such as the lauryl amine hydrochloride and F127.^[^ ^73^ ^]^ The smaller particles of Nb_2_O_5_ were observed with the increase of pH.^[^ ^74^ ^]^ Furthermore, other methods were developed for the synthesis of Nb_2_O_5_ particles. For instance, the supercritical‐CO_2_‐assisted approach was introduced into catalyst preparation.^[^ ^75^ ^]^ The hydrolyzed mixture of NbCl_5_ dissolved in ethanol solution and aged in the supercritical CO_2_ under 80 °C for 3 h. After the calcination under 200 °C, amorphous Nb_2_O_5_ particles with a high surface area (≈340 m^2^ g^−1^) were obtained. ^[^ ^75^ ^]^ In addition, the ball‐milling process was reported for the low‐temperature synthesis. Mixed Nb_2_O_5_ and Na_2_CO_3_ were formed by the reaction of NbCl_5_ and Na_2_CO_3_. ^[^ ^76^ ^]^ The molar ratio of NbCl_5_ to Na_2_CO_3_ and calcination temperature were controlled to inhibit the generation of unwanted niobates.^[^ ^76^ ^]^ The additives, such as urea and melamine, acted as the fuel and template to fabricate TT‐Nb_2_O_5_ particles in the calcination process.^[^ ^12^, ^77^ ^]^ Furthermore, the as‐synthesized H‐Nb_2_O_5_ particles can be treated under laser pulses to prepare amorphous Nb_2_O_5_, T‐Nb_2_O_5_, and TT‐Nb_2_O_5_, realizing the reversible transformation of crystal structures.^[^ ^78^ ^]^

Other morphologies of Nb_2_O_5_ catalysts were also reported, such as the bulk, octahedron, hollow structure, and others.^[^ ^79^ ^]^ The synthetic methods of these morphologies were partly similar to that of Nb_2_O_5_ particles under different conditions. For instance, the Nb_2_O_5_ particles can be further calcinated to prepare the bulk counterpart.^[^ ^75^ ^]^ Besides, the resorcinol, formaldehyde, and ammonium niobate oxalate were utilized in the hydrothermal process for the synthesis of Nb_2_O_5_@polymer materials, which were further calcinated to remove the polymer and obtain the hollow Nb_2_O_5_ microspheres.^[^ ^80^ ^]^

### 3.2 Synthesis of Nb_2_O_5_ Catalysts with Other Species

#### 3.2.1 Doped Nb_2_O_5_ Catalysts

**Figure 5:**
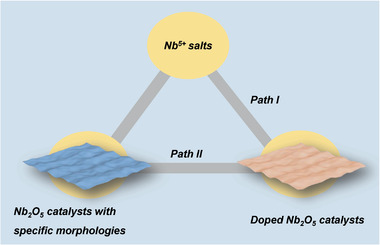
The synthesis of doped Nb_2_O_5_ catalysts.

Because of the wide bandgap of Nb_2_O_5_ (≈3.0‐3.4 eV), the strategies by introducing doped atoms are applied to enhance the optical absorption ability, which was confirmed by experiments and first‐principles calculations.^[^ ^81^ ^]^ The synthetic methodologies can be classified into two approaches: a) synthesis from the mixture of additives and columbic precursors, and b) post‐treatment of as‐synthesized Nb_2_O_5_ catalysts (**Figure** **5**). For instance, ethanol and acetic acid acted as the carbon sources and the solvent to prepare the carbon‐doped Nb_2_O_5_ (C–Nb_2_O_5_) in the solvothermal process (Figure 5, path I).^[^ ^82^ ^]^ Similarly, the niobium ethoxide and NbCl_5_ were dispersed in a mixed solution of alcohol and nitrogenous additives in the solvothermal process, leading to the formation of nitrogen‐doped Nb_2_O_5_ (N–Nb_2_O_5_).^[^ ^33^, ^83^ ^]^ Besides, N–Nb_2_O_5_ can be obtained from the calcination of niobium salts with urea, melamine, and ammonium chloride.^[^ ^12^, ^84^ ^]^ A series of rare‐earth (Er, Eu, Pr, Tm, and Yb), Ag, Fe, Mo, Pd, Sr, W, Y, Zn, and Zr doped Nb_2_O_5_ materials were also synthesized from the calcination of mixing the niobium salts with other metal additives.^[^ ^77^, ^85^ ^]^ Additionally, the alkali metal doped Nb_2_O_5_ materials were also fabricated by the electrochemical approach. The Nb foils were oxidized at a pulsed current while the alkali metal ions in the electrolytes were feasible to interact with NbO*_x_*, resulting in the generation of M–Nb_2_O_5_ (M = Li, Na, K, Rb, and Cs).^[^ ^86^ ^]^

Moreover, as‐synthesized Nb_2_O_5_ can be further treated (Figure 5, path II). Mesoporous Nb_2_O_5_ and citric acid were utilized to synthesize the C–Nb_2_O_5_ under the calcination at 400 °C.^[^ ^73^ ^]^ The Nb_2_O_5_ was treated with NH_3_ gas to prepare N–Nb_2_O_5_ under 400–600 °C.^[^ ^87^ ^]^ In addition, the N–Nb_2_O_5_ was also synthesized from the calcination of porous Nb_2_O_5_ with urea.^[^ ^19^, ^50^, ^88^ ^]^ If urea is replaced by thiourea, the generation of sulfur‐doped Nb_2_O_5_ (S–Nb_2_O_5_) can be observed.^[^ ^73^ ^]^ Furthermore, N, S codoped Nb_2_O_5_ was obtained when the ratio of thiourea to Nb_2_O_5_ increased from 0.37 to 1.^[^ ^89^ ^]^ Other metal atoms, like Mo atoms, can be introduced into the Nb_2_O_5_ lattices, which were synthesized from the hydrothermal process of ultrathin Nb_2_O_5_ nanosheets and ammonium molybdate.^[^ ^90^ ^]^

#### 3.2.2 Metal Species Supported on Nb_2_O_5_ (M/Nb_2_O_5_) Catalysts

Diverse metals, metal oxides, and metal salts are utilized in the synthesis of M/Nb_2_O_5_. For instance, Pt and Ag_2_O powders were mixed with Nb_2_O_5_ to prepare the Pt/Nb_2_O_5_ and Ag/Nb_2_O_5_, respectively.^[^ ^91^ ^]^ Accompanied by the development in nanotechnology, the size of metal particles was precisely controlled by the colloidal method, which can be further applied in the synthesis of M/Nb_2_O_5_.^[^ ^48^, ^92^ ^]^ For instance, ≈7.0 nm Pd nanoparticles were protected by the ligand, oleylamine, or oleic acid and introduced into the Nb_2_O_5_ suspension with the assistance of hexanes. The ligands on the Pd species can be further removed by calcination.^[^ ^92d^ ^]^

Metal salts were applied in the synthesis of M/Nb_2_O_5_ by the wet chemistry methods.^[^ ^93^ ^]^ The Au/Nb_2_O_5_, Ir/Nb_2_O_5_, Rh/Nb_2_O_5_, Ru/Nb_2_O_5_, Pd/Nb_2_O_5_, and Pt/Nb_2_O_5_ were synthesized by the incipient wetness impregnation method.^[^ ^93^ ^]^ The heteroatoms in the precursor, such as nitrogen and chlorine atoms, are reasonably removed by the calcination process.^[^ ^93a^ ^]^ Whereas, the aggregation of metal species to nanoparticles was observed, ascribed to the heat treatment. The deposition–precipitation method was also developed to produce M/Nb_2_O_5_, following the calcination under lower temperature (≈300 °C).^[^ ^8c^ ^]^ In the preparation of catalysts, Au^3+^ ions were deposited on the Nb_2_O_5_ surface with the assistance of urea or ammonium hydroxide. The average diameter of Au nanoparticles was ≈5 nm after calcination.^[^ ^8c^ ^]^ The H_2_ can be replaced by NaBH_4_ or hydrazine, which is an effective reductant for RuCl_3_ and Pd(acac)_2_ to Ru/Nb_2_O_5_ and Pd/Nb_2_O_5_ without heat treatment.^[^ ^94^ ^]^ Instead of NaBH_4_ and hydrazine, the reductive electrons can be directly generated from Nb_2_O_5_ under UV light irradiation.^[^ ^4a^ ^]^ The Au/Nb_2_O_5_, Pt/Nb_2_O_5_, and Pd/Nb_2_O_5_ were fabricated by this approach. ^[^ ^95^ ^]^ Meanwhile, the photogenerated holes were captured by sacrificial agents, such as ethanol or isopropanol.^[^ ^95^ ^]^ Besides, the electrostatic adsorption was available to prepare the highly dispersed metal nanoparticles supported on Nb_2_O_5_ under room temperature, attributed to the difference in point of zero charges (PZCs) between the metal ions and Nb_2_O_5_ at the same pH. Thus, Ag/Nb_2_O_5_ can be synthesized via the interaction between the Ag(NH_3_)_2_^+^ and Nb_2_O_5_ at high pH (>5).^[^ ^96^ ^]^

#### 3.2.3 Composited Nb_2_O_5_ Catalysts

**Figure 6:**
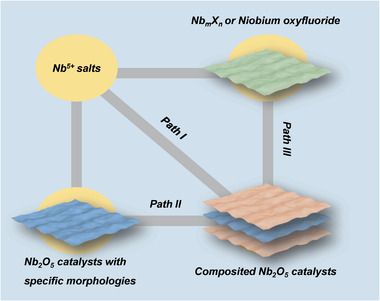
The synthesis of composited Nb_2_O_5_ catalysts.

The Nb_2_O_5_ can be modified by other metal oxides, metal sulfides, metal carbides, carbon materials, carbon nitride (g‐C_3_N_4_), and black phosphorus (BP) to enhance its activity.^[^ ^13^, ^97^ ^]^ As‐prepared Nb_2_O_5_, NbC_2_, Nb_3_O_7_F, and niobium salts were reported in the synthesis of composited Nb_2_O_5_ catalysts (**Figure** **6**).

Different Nb salts were reported in the synthesis of composited Nb_2_O_5_ photocatalysts (Figure 6, path I).^[^ ^98^ ^]^ The methods mentioned in the preparation of pure phase Nb_2_O_5_ were also applied in the composited Nb_2_O_5_ photocatalysts. For instance, the CVD method was utilized to fabricate Nb_2_O_5_/SiO_2_.^[^ ^98^ ^]^ Besides, Nb precursors, like NbCl_5_, and other metal salts were cohydrolyzed and precipitated to prepare mixed metal oxide particles.^[^ ^99^ ^]^ Especially, ≈25–51 nm Nb_2_O_5_ nanocrystals were observed on the surface of ZnO nanorods, while ≈3–5 nm Nb_2_O_5_ microspheres were fabricated on the g‐C_3_N_4_.^[^ ^99e,g^ ^]^ This phenomenon can be ascribed to P123, which were conducive to the stabilization and dispersion of Nb micelles in the synthesis of Nb_2_O_5_/g‐C_3_N_4_.^[^ ^99g^ ^]^

In addition, as‐prepared Nb_2_O_5_ can be ground directly with TiO_2_, SrNb_2_O_6_, Bi_2_O_3_, and GO to prepare the TiO_2_/Nb_2_O_5_, SrNb_2_O_6_/Nb_2_O_5_, and GO/Bi_2_O_3_/Nb_2_O_5_, respectively.^[^ ^100^ ^]^ Further calcination was conducted to improve the interaction between the Nb_2_O_5_ and other components (Figure 6, path II).^[^ ^101^ ^]^ Besides, as‐prepared Nb_2_O_5_ was also dispersed in solutions, such as isopropanol or tetrahydrofuran, to enhance the contact with the TiO_2_, BP, and C_60_.^[^ ^97^, ^102^ ^]^ In addition, the metal salt precursors were introduced into Nb_2_O_5_ suspension instead of as‐synthesized metal oxide or metal sulfide.^[^ ^103^ ^]^ The ZnO/Nb_2_O_5_ and CdS/Nb_2_O_5_ were prepared from Zn(NO_3_)_2_ and CdCl_2_ by the impregnation approach, respectively.^[^ ^104^ ^]^ The metal precursors, like TiCl_4_, were hydrolyzed by the addition of NH_3_·H_2_O and deposited on the Nb_2_O_5_ to obtain the TiO_2_/Nb_2_O_5_.^[^ ^103a^ ^]^ Besides, CdS/Nb_2_O_5_/N‐GO was obtained from Nb_2_O_5_ by the deposition of CdS in the hydrothermal process. Especially, electrostatic adsorption was also reported to fabricate composited Nb_2_O_5_ catalysts.^[^ ^13^, ^105^ ^]^ In principle, the PZC can be utilized to screen materials that exhibit positive or negative surface charges, which are opposite to that on the Nb_2_O_5_ surface at the same pH value. Fortunately, SiO_2_ and g‐C_3_N_4_ as the potential candidates were reported.^[^ ^13^, ^105^ ^]^ Positive charges originated from amino groups that were exposed on the g‐C_3_N_4_ surface in a pH range of 3–4. Meanwhile, the Nb_2_O_5_ surface is electronegative, ascribed to the presence of surface hydroxyl groups.^[^ ^13^ ^]^ Compared to the impregnation approach, this method is available to prepare highly dispersed components on Nb_2_O_5_, due to the adsorption equilibrium. Excess g‐C_3_N_4_ are possibly removed by washing and filtration, which differ from the drying treatment in the impregnation approach.^[^ ^13^ ^]^ Furthermore, isolated species on Nb_2_O_5_ may be obtained by this method with precise control of precursor concentration, pH, and temperature.

Additionally, as‐synthesized NbC_2_ and Nb_3_O_7_F were also utilized to prepare corresponding Nb_2_O_5_‐based catalysts (Figure 6, path III). The Nb_2_O_5_/C/Nb_2_C and Nb_3_O_7_F/Nb_2_O_5_ photocatalysts were obtained by one‐step calcination.^[^ ^97^, ^106^ ^]^ The formation of Nb_3_O_7_F/Nb_2_O_5_ was ascribed to the decomposition of Nb_3_O_7_F to Nb_2_O_5_ when the temperature is higher than 400 °C.^[^ ^106^ ^]^ Similarly, the observed Nb_2_O_5_ supported on Nb_2_C was due to the reaction between Nb_2_C and CO_2_ under 850 °C.^[^ ^97d^ ^]^ Considering the thermal reaction, a series of composited photocatalysts with different Nb_2_O_5_ fractions are feasibly prepared, using the Nb_2_C, NbN, NbSe_2_, NbS_2_, and Nb_3_O_7_F.^[^ ^107^ ^]^

## 4 Application of Nb_2_O_5_‐Based Photocatalysts

### 4.1 Photodegradation of Pollutants

Nowadays, Nb_2_O_5_‐based photocatalysts were widely reported in the photocatalytic photodegradation of pollutants (**Table** **1**).^[^ ^108^ ^]^ The hydrocarbons and chlorinated hydrocarbons (Table 1, Nos. 1–5), phenols (Table 1, Nos. 6–16), aldehydes (Table 1, No. 17), organic acids (Table 1, Nos. 18–26), ester (Table 1, No. 27), organic dyes, and pesticides (Table 1, No. 28–145) are oxidized on Nb_2_O_5_‐based photocatalysts.^[^ ^77^, ^92^, ^109^ ^]^ In these processes, the wavelength (*λ*) of light sources is vital in photocatalysis, due to the limited absorption edge of photocatalysts.^[^ ^8e^ ^]^ For instance, pure Nb_2_O_5_ exhibits large *E*_g_ and is active under UV light irradiation (*λ* < 400 nm).^[^ ^4a^ ^]^ Accordingly, the black lamp, Xe lamp, and Hg lamp are available light sources.^[^ ^108^, ^110^ ^]^ Considering that ≈4% of the total solar spectrum is UV fraction, the strategies were developed to enhance the utilization efficiency of sunlight.^[^ ^8e^ ^]^ Catalyst modification by dopant, surface metal species, and other semiconductors were useful to extend the optical absorption edge of Nb_2_O_5_.^[^ ^5b^ ^]^ Thus, photodegradation of pollutants were also reported over Nb_2_O_5_‐based photocatalysts under visible light irradiation from other sources, including the fluorescent lamps, halide lamp, white LEDs, solar simulator, and sunlight.^[^ ^77^, ^99^, ^109^, ^111^ ^]^ In these processes, the ultraviolet filter can be utilized to eliminate the effect of UV light. Especially, the degradation of RhB was driven under visible light over pure Nb_2_O_5_ without the limitation of its bandgap energy (Table 1, No. 101). This process is ascribed to the dye‐sensitized photocatalysis, in which the RhB molecules adsorbed on the Nb_2_O_5_ surface are excited by 440 nm light irradiation.^[^ ^109^, ^112^ ^]^ The electrons transfer from the highest occupied molecular orbital (HOMO) to lowest unoccupied molecular orbital (LUMO) of RhB molecules and inject into the Nb_2_O_5_ conduction band, which induce the generation of active species for the succedent mineralization of the organic pollutant.^[^ ^112^ ^]^

**Table 1:** Recent advances in the photodegradation of pollutants over Nb_2_O_5_‐based photocatalysts

No.	Catalysts	Pollutants	Light sources	Reaction temperature [°C]	Degradation rate	Refs.
1	N‐TiO_2_–Nb_2_O_5_	Benzene, toluene, and xylene	46 W black lamp	25	10 min^−1^	^[^ ^108^ ^]^
2	N–Nb_2_O_5_	Toluene	Xe lamp	n.m.^a)^	≈10% (60 min)	^[^ ^110a^ ^]^
3	Pt/Nb_2_O_5_	Ethylene	Xe lamp	n.m.	0.94 min^−1^	^[^ ^95c^ ^]^
4	T‐Nb_2_O_5_ nanotubes	Trichloro‐ethylene	UV light	n.m.	100% (15 min)	^[^ ^23c^ ^]^
5	Nb_2_O_5_/TiO_2_	1,4‐dichlorobenzene	150 W Xe lamp	n.m.	≈60% (10 min)	^[^ ^118^ ^]^
6	TT‐Nb_2_O_5_ particles	2‐chlorophenol	400 W halide lamp (350–700 nm)	30	0.13 h^−1^	^[^ ^77^ ^]^
7	Nb_2_O_5_ nanorods/graphene	4‐chlorophenol	300 W Xe lamp (420–780 nm)	n.m.	≈95% (210 min)	^[^ ^119^ ^]^
8	Carbon xerogel/Nb_2_O_5_/TiO_2_	4‐chlorophenol	300 W lamp	25	0.0078 min^−1^	^[^ ^120^ ^]^
9	WO_3_/Nb_2_O_5_	4‐nitrophenol	125 W Hg lamp	27	4.6 s^−1^	^[^ ^121^ ^]^
10	CeO_2_/Nb_2_O_5_	Phenol	UV light	n.m.	90% (150 min)	^[^ ^122^ ^]^
11	Nb_2_O_5_	Phenol	UV light	n.m.	14% (15 min)	^[^ ^123^ ^]^
12	Nb_2_O_5_–Pr_6_O_11_	Phenol	6 W Hg lamp	n.m.	2.5 × 10^−6^ m s^−1^	^[^ ^124^ ^]^
13	Nb_2_O_5_–ZnS	Phenol	8 W Hg lamp	n.m.	58% (15 min)	^[^ ^125^ ^]^
14	Nb_2_O_5_/ZnO rods	Phenol	Sunlight	n.m.	100% (40 min)	^[^ ^99e^ ^]^
15	Nb_2_O_5_/ZnO	Phenol	15 W Hg lamp	n.m.	100% (60 min)	^[^ ^110b^ ^]^
16	Sr–Nb_2_O_5_	2‐chlorophenol	400 W halide lamp (350–700 nm)	30	0.58 h^−1^	^[^ ^77^ ^]^
17	Nb_2_O_5_–TiO_2_	Acetaldehyde	Xe lamp (350–700 nm)	r.t.^b)^	0.0139 min^−1^	^[^ ^126^ ^]^
18	Amorphous Nb_2_O_5_ particles	Acetic acid	400 W Hg lamp (*λ* > 300 nm)	25	53 µmol h^−1^ g^−1^	^[^ ^70^ ^]^
19	Pt–TiO_2_–Nb_2_O_5_	Ketoprofen	UV LEDs	n.m.	0.174 min^−1^	^[^ ^102a^ ^]^
20	Nb_2_O_5_	Caffeic acid	White LED	25	≈55% (180 min)	^[^ ^127^ ^]^
21	Pt–TiO_2_–Nb_2_O_5_	Diclofenac	UV LEDs	n.m.	0.446 min^−1^	^[^ ^102a^ ^]^
22	Nb_2_O_5_	Oxalic acid	300 W Xe lamp	25	40% (240 min)	^[^ ^128^ ^]^
23	Mesoporous Nb_2_O_5_	Terephthalic acid	400 W Hg lamp	25	100% (60 min)	^[^ ^129^ ^]^
24	Nb_2_O_5_/C_3_N_4_	Tetracycline hydrochloride	250 W Xe lamp (*λ* > 420 nm)	25	76% (150 min)	^[^ ^116^ ^]^
25	g‐C_3_N_4_–mesoporous Nb_2_O_5_	Tetracycline hydrochloride	300 W Xe lamp (*λ* > 420 nm)	n.m.	76% (60 min)	^[^ ^99h^ ^]^
26	Zn–Nb_2_O_5_	Caffeic acid	15 W UV light	r.t.	80% (180 min)	^[^ ^85j^ ^]^
27	Fe_2_O_3_/Nb_2_O_5_	Ethyl 4‐hydroxy‐benzoate	300 W Xe lamp (*λ* > 400 nm)	n.m.	≈55% (12 h)	^[^ ^130^ ^]^
28	NiO–Nb_2_O_5_	Indigo carmine	20 W UV light	n.m.	≈90% (90 min)	^[^ ^131^ ^]^
29	Zr–Nb_2_O_5_	Indigo carmine	400 W halide lamp (350–700 nm)	30	0.52 h^−1^	^[^ ^77^ ^]^
30	Nb_2_O_5_	Indigo carmine	125 W Hg lamp	n.m.	100% (25 min)	^[^ ^132^ ^]^
31	TT‐Nb_2_O_5_ particles	Indigo carmine	400 W halide lamp (350–700 nm)	30	0.29 h^−1^	^[^ ^77^ ^]^
32	TiO_2_/Nb_2_O_5_	Indigo carmine	36 W UV lamp (200–400 nm)	n.m.	≈87% (120 min)	^[^ ^103a^ ^]^
33	Nb_2_O_5_/cellulose acetate	Indigo carmine	125 W Hg lamp	n.m.	≈99% (120 min)	^[^ ^133^ ^]^
34	Nb_2_O_5_ hollow spheres	Indigo carmine	100 W Hg lamp	n.m.	≈90% (80 min)	^[^ ^109a^ ^]^
35	g‐C_3_N_4_/Nb_2_O_5_	Malachite green	150 W white LED light	n.m.	100% (90 min)	^[^ ^109b^ ^]^
36	Amorphous Nb_2_O_5_ particles	Malachite green	400 W Hg lamp	25	0.014 min^−1^	^[^ ^65a^ ^]^
37	Ag/TiO_2_/Nb_2_O_5_	Malachite green	Visible light	25	100% (20 min)	^[^ ^92b^ ^]^
38	TT‐Nb_2_O_5_ particles	Orange G	400 W halide lamp (350–700 nm)	30	0.13 h^−1^	^[^ ^77^ ^]^
39	Sr–Nb_2_O_5_	Orange G	400 W halide lamp (350–700 nm)	30	0.20 h^−1^	^[^ ^77^ ^]^
40	TT‐Nb_2_O_5_ particles	MB^c)^	400 W halide lamp (350–700 nm)	30	0.19 h^−1^	^[^ ^77^ ^]^
41	Sr–Nb_2_O_5_	MB	400 W halide lamp (350–700 nm)	30	0.60 h^−1^	^[^ ^77^ ^]^
42	Nb_2_O_5_	MB	100 W Hg lamp	25	≈90% (120 min)	^[^ ^109c^ ^]^
43	TT‐Nb_2_O_5_ nanorods	MB	UV light	r.t.	0.0733 min^−1^	^[^ ^33a^ ^]^
44	TT‐Nb_2_O_5_ nanorods	MB	500 W Hg lamp	r.t.	≈93% (150 h)	^[^ ^34c^ ^]^
45	Mesoporous Nb_2_O_5_	MB	250 W Xe lamp	30	0.014 min^−1^	^[^ ^134^ ^]^
46	TT‐Nb_2_O_5_ spheres	MB	500 W Hg lamp	r.t.	≈73% (150 h)	^[^ ^34c^ ^]^
47	TT‐Nb_2_O_5_ fibers	MB	500 W Hg lamp	n.m.	96% (50 min)	^[^ ^68^ ^]^
48	H‐Nb_2_O_5_ particles	MB	15 W UV light	30	0.198 h^−1^	^[^ ^135^ ^]^
49	T‐Nb_2_O_5_ particles	MB	UV lamp	r.t.	60% (60 min)	^[^ ^74^ ^]^
50	Mixed phase Nb_2_O_5_ particles	MB	UV lamp	n.m.	95% (120 min)	^[^ ^136^ ^]^
51	Nb_2_O_5_ nanofibers	MB	300 W Xe lamp	n.m.	45% (120 min)	^[^ ^137^ ^]^
52	Nb_2_O_5_ fibers	MB	100 W Hg lamp	n.m.	0.025 min^−1^	^[^ ^138^ ^]^
53	Nb_2_O_5_	MB	300 W Hg lamp	r.t.	70% (480 min)	^[^ ^139^ ^]^
54	Nb_2_O_5_ nanoparticles	MB	150 W Hg lamp	25	90% (150 min)	^[^ ^140^ ^]^
55	Nb_2_O_5_	MB	450 W solar simulator	n.m.	90% (20 min)	^[^ ^141^ ^]^
56	Nb_2_O_5_	MB	24 W lamps	25	40% (80 min)	^[^ ^142^ ^]^
57	Nb_2_O_5_	MB	UV light	n.m.	40% (300 min)	^[^ ^143^ ^]^
58	N–Nb_2_O_5_	MB	500 W Xe lamp	25	40% (240 min)	^[^ ^87^ ^]^
59	Mo–Nb_2_O_5_ W–Nb_2_O_5_	MB	UV light	25	n.m.	^[^ ^85h^ ^]^
60	Pd‐xerogel/Nb_2_O_5_	MB	Visible light	r.t.	30% (300 min)	^[^ ^85i^ ^]^
61	Nb_2_O_5_/TiO_2_	MB	400 W Xe lamp	n.m.	0.072 min^−1^	^[^ ^101a^ ^]^
62	Nb_2_O_5_–TiO_2_	MB	UV light	r.t.	100% (240 min)	^[^ ^99a^ ^]^
63	Nb_2_O_5_/TiO_2_	MB	15 W fluorescent lamps (390–720 nm)	n.m.	84% (150 min)	^[^ ^111a^ ^]^
64	Nb_2_O_5_/NaX zeolite	MB	80 W Xe lamp	25	60% (300 min)	^[^ ^144^ ^]^
65	Nb_2_O_5_/MCM‐41	MB	15 W UV lamp	n.m.	60% (60 min)	^[^ ^145^ ^]^
66	Nb_2_O_5_/Nb_3_O_7_F	MB	Xe lamp (380–780 nm)	r.t.	100% (80 min)	^[^ ^106^ ^]^
67	*α*‐Fe_2_O_3_/Nb_2_O_5_	MB	300 W simulated solar irradiation	n.m.	80% (120 min)	^[^ ^111b^ ^]^
68	CdS@Nb_2_O_5_	MB	125 W Hg lamp	n.m.	80% (180 min)	^[^ ^97b^ ^]^
69	Carbon xerogel–Nb_2_O_5_	MB	Visible light	r.t.	30% (300 min)	^[^ ^146^ ^]^
70	Carbon xerogel–Nb_2_O_5_	MB	Visible light	25	60% (300 min)	^[^ ^147^ ^]^
71	Nb_2_O_5_/tannin‐formaldehyde xerogel	MB	300 W UV lamp (200–280 nm)	25	100% (90 min)	^[^ ^148^ ^]^
72	Carbon xerogel–Nb_2_O_5_	MB	300 W simulated solar	r.t.	80% (300 min)	^[^ ^149^ ^]^
73	CeO_2_/Nb_2_O_5_	MB	UV light	n.m.	98% (150 min)	^[^ ^122^ ^]^
74	g‐C_3_N_4_/Nb_2_O_5_	MB	UV light	18	90% (210 min)	^[^ ^99f^ ^]^
75	TT‐Nb_2_O_5_ spheres	MB	Xe lamp (*λ* > 380 nm)	r.t.	90% (90 min)	^[^ ^71a^ ^]^
76	Nb_2_O_5_–graphene	MB	UV light	n.m.	99% (5 min)	^[^ ^150^ ^]^
77	T‐Nb_2_O_5_ nanowires	MB	100 W mercury lamp	r.t.	95% (150 min)	^[^ ^37a^ ^]^
78	Nb_2_O_5_–C_60_	MB	UV lamp	n.m.	97% (5 min)	^[^ ^102b^ ^]^
79	Ag/Nb_2_O_5_	MB	500 W mercury lamp	n.m.	0.0108 min^−1^	^[^ ^151^ ^]^
80	TiO_2_/Nb_2_O_5_/r‐GO	MB	300 W Xe lamp	24–28	97% (240 min)	^[^ ^152^ ^]^
81	MnO_2_/Nb_2_O_5_/carbon clusters	MB	Visible light (*λ* > 460 nm)	n.m.	n.m.	^[^ ^153^ ^]^
82	N‐TiO_2_–Nb_2_O_5_	MB	13 W fluorescent lamp	n.m.	66% (180 min)	^[^ ^154^ ^]^
83	Nb_2_O_5_ nanowires	MB	UV light	r.t.	92% (120 min)	^[^ ^155^ ^]^
84	Nb_2_O_5_ nanoplates	MB	100 W Hg lamp	r.t.	≈92% (60 min)	^[^ ^156^ ^]^
85	Ag/TiO_2_/Nb_2_O_5_	MO^d)^	Visible light	25	12% (120 min)	^[^ ^92b^ ^]^
86	r‐GO/SnO_2_/Nb_2_O_5_/TiO_2_	MO	300 W Xe lamp (*λ* > 400 nm)	30–35	95% (120 min)	^[^ ^109f^ ^]^
87	TiO_2_/Nb_2_O_5_/r‐GO	MO	300 W Xe lamp	30–35	93% (240 min)	^[^ ^152^ ^]^
88	T‐Nb_2_O_5_ nanowires	MO	100 W Hg lamp	r.t.	70% (150 min)	^[^ ^37a^ ^]^
89	Nb_2_O_5_ nanofibers	MO	300 W Hg lamp	r.t.	62% (180 min)	^[^ ^157^ ^]^
90	Nb_2_O_5_	MO	400 W Hg lamp	r.t.	78% (80 min)	^[^ ^158^ ^]^
91	Nb_2_O_5_	MO	Sunlight	25	95% (60 min)	^[^ ^159^ ^]^
92	Ag_3_PO_4_/Nb_2_O_5_	MO	600 W Xe lamp	n.m.	100% (25 min)	^[^ ^160^ ^]^
93	Nb_2_O_5_@G nanofibers	MO	400 W metal‐halide lamp (*λ* > 380 nm)	n.m.	0.547 h^−1^	^[^ ^161^ ^]^
94	Nb_2_O_5_/SrNb_2_O_6_	MO	300 W Hg lamp	n.m.	95% (40 min)	^[^ ^100b^ ^]^
95	Nb_2_O_5_/SrNb_2_O_6_	MO	500 W Hg lamp	n.m.	≈95% (28 min)	^[^ ^162^ ^]^
96	T‐Nb_2_O_5_ particles	RhB^e)^	UV light	25	61% (120 min)	^[^ ^163^ ^]^
97	TT‐Nb_2_O_5_ particles	RhB	UV light	18	0.00 757 min^−1^	^[^ ^164^ ^]^
98	TT‐Nb_2_O_5_ particles	RhB	UV light	n.m.	100% (60 min)	^[^ ^165^ ^]^
99	Amorphous Nb_2_O_5_ particles	RhB	5 W white LED light	n.m.	96% (70 min)	^[^ ^166^ ^]^
100	Flowerlike T‐Nb_2_O_5_	RhB	300 W Hg lamp	r.t.	100% (90 min)	^[^ ^167^ ^]^
101	T‐Nb_2_O_5_ spheres	RhB	300 W Xe lamp (*λ* > 420 nm)	n.m.	0.2099 min^−1^	^[^ ^112^ ^]^
102	Nb_2_O_5_	RhB	UV light	25	78% (120 min)	^[^ ^168^ ^]^
103	Nb_2_O_5_ microflowers	RhB	50 W Hg lamp	n.m.	0.238 min^−1^	^[^ ^169^ ^]^
104	Nb_2_O_5_	RhB	8 W Hg lamp	r.t.	0.0669 min^−1^	^[^ ^170^ ^]^
105	Nb_2_O_5_ nanoplates	RhB	100 W Hg lamp	r.t.	≈98% (60 min)	^[^ ^156^ ^]^
106	C‐modified Nb_2_O_5_	RhB	500 W tungsten halogen lamp	n.m.	100% (180 min)	^[^ ^171^ ^]^
107	C–Nb_2_O_5_	RhB	Xe lamp	n.m.	100% (30 min)	^[^ ^82a^ ^]^
108	N–Nb_2_O_5_	RhB	300 W Xe lamp (*λ* > 400 nm)	n.m.	100% (15 min)	^[^ ^88^ ^]^
109	C, N‐modified Nb_2_O_5_	RhB	300 W Xe lamp (*λ* > 420 nm)	15	100% (40 min)	^[^ ^172^ ^]^
110	C, N‐modified Nb_2_O_5_	RhB	300 W Xe lamp (420–720 nm)	n.m.	0.13 572 min^−1^	^[^ ^173^ ^]^
111	N, S–Nb_2_O_5_	RhB	UV light	n.m.	92% (180 min)	^[^ ^89^ ^]^
112	N–HNb_3_O_8_	RhB	300 W Xe lamp (*λ* > 420 nm)	n.m.	98% (50 min)	^[^ ^174^ ^]^
113	C–Nb_2_O_5_	RhB	300 W Xe lamp (*λ* > 420 nm)	25	≈90% (30 min)	^[^ ^175^ ^]^
114	N–HNb_3_O_8_	RhB	300 W Xe lamp (*λ* > 420 nm)	n.m.	98% (50 min)	^[^ ^176^ ^]^
115	Au@void@Nb_2_O_5_	RhB	300 W Xe lamp (*λ* > 420 nm)	15	100% (140 min)	^[^ ^92a^ ^]^
116	Nb_2_O_5_/Pd@SBA‐15	RhB	UV light	r.t.	97% (210 min)	^[^ ^177^ ^]^
117	Nb_2_O_5_/FTO	RhB	300 W Hg lamp	n.m.	0.01 212 min^−1^	^[^ ^178^ ^]^
118	BiOCl/Nb_2_O_5_/Bi_4_NbO_8_Cl	RhB	300 W Hg lamp	n.m.	99% (40 min)	^[^ ^179^ ^]^
119	Nb_2_O_5_–g‐C_3_N_4_/graphene aerogel	RhB	300 W Xe lamp (*λ* > 420 nm)	n.m.	95% (100 min)	^[^ ^180^ ^]^
120	BiNb_5_O_14_/Nb_2_O_5_	RhB	500 W Xe lamp (*λ* > 420 nm)	n.m.	61% (60 min)	^[^ ^181^ ^]^
121	Nb_2_O_5_–WO_3_	RhB	125 W Hg lamp	n.m.	≈70% (100 min)	^[^ ^182^ ^]^
122	TT‐Nb_2_O_5_ particles	RhB	UV light	18	0.00 323 min^−1^	^[^ ^109e^ ^]^
123	g‐C_3_N_4_/Nb_2_O_5_	RhB	15 W fluorescent lamps	18	0.0202 min^−1^	^[^ ^13^ ^]^
124	T‐Nb_2_O_5_ nanowires	RhB	100 W Hg lamp	r.t.	95% (150 min)	^[^ ^37a^ ^]^
125	C–Nb_2_O_5_	RhB	300 W Xe lamp (*λ* > 420 nm)	n.m.	100% (30 min)	^[^ ^82b^ ^]^
126	TT‐Nb_2_O_5_ nanowires	RhB	500 W Xe lamp (*λ* > 420 nm)	n.m.	0.047 min^−1^	^[^ ^183^ ^]^
127	g‐C_3_N_4_–mesoporous Nb_2_O_5_	RhB	300 W Xe lamp (*λ* > 420 nm)	n.m.	98% (180 min)	^[^ ^99h^ ^]^
128	Zn–Nb_2_O_5_	RhB	15 W UV light	r.t.	90% (180 min)	^[^ ^85j^ ^]^
129	Zn–C/Nb_2_O_5_	RhB	Visible light	n.m.	100% (80 min)	^[^ ^184^ ^]^
130	Cd*_x_*Zn*_y_*S/Nb_2_O_5_	Violet	100 W fluorescent lamps	n.m.	0.054 min^−1^	^[^ ^104b^ ^]^
131	r‐GO/SnO_2_/Nb_2_O_5_/TiO_2_	Violet	300 W Xe lamp (*λ* > 400 nm)	30–35	98% (120 min)	^[^ ^109f^ ^]^
132	TT‐Nb_2_O_5_ particles	Atrazine	UV light	18	0.0124 min^−1^	^[^ ^164^ ^]^
133	TT‐Nb_2_O_5_ particles	Atrazine	UV light	18	0.03 min^−1^	^[^ ^109e^ ^]^
134	Nb_2_O_5_	Basic red‐2	400 W Hg lamp	25	94% (120 min)	^[^ ^185^ ^]^
135	Mesoporous TT‐Nb_2_O_5_ particles	Methylviologen	125 W Hg lamp	25	0.041 min^−1^	^[^ ^186^ ^]^
136	Fe_2_O_3_/Nb_2_O_5_	Triclosan	125 W Hg lamp	25	0.069 min^−1^	^[^ ^187^ ^]^
137	Nb_2_O_5_/bentonite clay	Blue 19	125 W Hg lamp	25	98% (120 min)	^[^ ^188^ ^]^
138	Nb_2_O_5_/activated charcoal	Blue 5G	250 W Hg lamp	28	≈94 (300 min)	^[^ ^189^ ^]^
139	ZnO/Nb_2_O_5_	Bromophenol blue	400 W Hg lamp	25	0.030 min^−1^	^[^ ^104a^ ^]^
140	Nb_2_O_5_/ZnAl‐LDH	Congo red	300 W Xe lamp (*λ* > 420 nm)	n.m.	≈85% (390 min)	^[^ ^190^ ^]^
141	Nb_2_O_5_/Bi_2_WO_6_	Dibenzo‐thiophene	5 W LED lamps	r.t.	99% (120 min)	^[^ ^191^ ^]^
142	Nb_2_O_5_	Reactive blue 59	400 W Hg lamp	n.m.	89% (150 min)	^[^ ^192^ ^]^
143	TT‐Nb_2_O_5_ spheres	Rose bengal	Xe lamp (*λ* > 380 nm)	r.t.	60% (180 min)	^[^ ^71a^ ^]^
144	g‐C_3_N_4_/Nb_2_O_5_	Amiloride	15 W fluorescent lamps	18	0.0137 min^−1^	^[^ ^13^ ^]^
145	HNb_3_O_8_ nanosheets	Bromocresol green	Hg lamp	20–25	≈90% (45 min)	^[^ ^50e^ ^]^
146	Fe_2_O_3_/Nb_2_O_5_	Paper wastewater	205 W Hg lamp	r.t.	0.061 h^−1^	^[^ ^113a^ ^]^
147	Ag_2_O/Nb_2_O_5_	Paper wastewater	205 W Hg lamp	r.t.	0.094 h^−1^	^[^ ^113a^ ^]^
148	Nb_2_O_5_	Textile wastewater	250 W Hg lamp	25	≈0.60 min^−1^	^[^ ^113b^ ^]^
149	Carbon black–Nb_2_O_5_	Textile wastewater	250 W Hg lamp	n.m.	≈41% (300 min)	^[^ ^113c^ ^]^
150	Ag/Nb_2_O_5_	Textile dyes	UV light bulb	n.m.	≈96% (24 h)	^[^ ^193^ ^]^
151	Nb_2_O_5_/NaX	Textile effluents	250 W Hg lamp	28	0.0033 min^−1^	^[^ ^194^ ^]^
152	Nb_2_O_5_/ZnO	Palm oil mill effluent	15 W UV lamp	n.m.	92% (240 min)	^[^ ^195^ ^]^
153	Nb_2_O_5_/ZnO	Palm oil mill efuent	15 W UV lamp	n.m.	92% (240 min)	^[^ ^196^ ^]^
154	Nb_2_O_5_	Petrol station wastewater	250 W Hg lamp	n.m.	≈35% (300 min)	^[^ ^197^ ^]^
155	Nb_2_O_5_–TiO_2_	Vinasse	Solar radiation	n.m.	≈55% (24 h)	^[^ ^100a^ ^]^
156	Nb_2_O_5_/TiO_2_	Cr(VI)	20 W UV lamp	n.m.	≈90% (180 min)	^[^ ^99c^ ^]^
157	TT‐Nb_2_O_5_ nanowires/carbon fiber	Cr(VI)	500 W UV light	n.m.	≈99% (60 min)	^[^ ^198^ ^]^
158	TT‐Nb_2_O_5_ nanorods/diatomite	Cr(VI)	500 W Hg lamp	r.t.	90% (60 min)	^[^ ^199^ ^]^
159	Porous TT‐Nb_2_O_5_	Cr(VI)	18 W UV light	n.m.	60% (120 min)	^[^ ^63b^ ^]^
160	Nb_2_O_5_	Cr(VI)	250 W Hg lamp	n.m.	≈90% (120 min)	^[^ ^200^ ^]^
161	N‐modified Nb_2_O_5_	Cr(VI)	Visible light (*λ* > 420 nm)	n.m.	≈80% (240 min)	^[^ ^117^ ^]^
162	CuO/Nb_2_O_5_	Cr(VI)	15 W UV lamps	18	23.10 min^−1^	^[^ ^201^ ^]^
163	Nb_2_O_5_@MIL‐125	Cr(VI)	990 W Xe lamp	25	≈99% (60 min)	^[^ ^202^ ^]^
164	TT‐Nb_2_O_5_ particles	*Escherichia coli*	Black light lamp	n.m.	0.034 min^−1^	^[^ ^203^ ^]^
165	Sr–Nb_2_O_5_	*Escherichia coli*	400 W halide lamp (350–700 nm)	30	0.12 min^−1^	^[^ ^77^ ^]^
166	Sr–Nb_2_O_5_	*Staphylococcus aureus*	400 W halide lamp (350–700 nm)	30	0.069 min^−1^	^[^ ^77^ ^]^

^a)^ Not mentioned

^b)^ Room temperature

^c)^ Methylene blue

^d)^ Methyl orange

^e)^ Rhodamine B.

**Figure 7:**
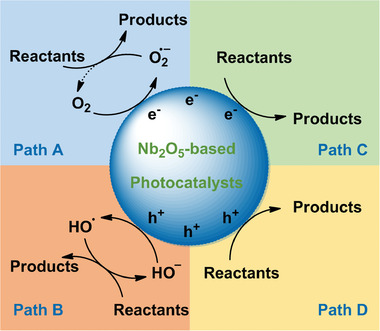
Possible reaction pathways over Nb_2_O_5_‐based photocatalysts.

Furthermore, the photodegradation of textile wastewater, palm oil mill effluent, petrol station wastewater, and vinasse was also reported (Table 1, Nos. 146–155).^[^ ^113^ ^]^ These results suggested the potential of Nb_2_O_5_‐based photocatalysts in practical applications. In these processes, the efficiency of catalysts is important in photocatalytic performance.^[^ ^5b^ ^]^ Generally, the degradation rate is a common criterion for the comparison of activity (Table 1). However, the degradation rate is related to the ratio of the moles of organic pollutants to the mass of catalyst, temperature, and intensity of the light source. For instance, a change of reaction temperature is beneficial to the separation of photogenerated electrons and holes.^[^ ^114^ ^]^ Increasing the intensity of the light source can improve the number of incident photons to enhance the reaction rate.^[^ ^115^ ^]^ Thus, it is a complicated and difficult process for the comparison of the activity results. The utilization of photogenerated electrons and holes over Nb_2_O_5_‐based photocatalysts can be used as another one criterion for the comparison of their activity. The organic pollutants can be degraded by the superoxide anions (•O_2_^−^), hydroxyl radicals (•OH), and photogenerated holes (h^+^), corresponding to the path A, path B, and path D in **Figure** **7**.^[^ ^13^, ^116^ ^]^ Therefore, the efficiency of electrons can be calculated by the ratio of products to the pollutants. However, the qualitative and quantitative analysis of obtained products is not always mentioned in the literature, leading to challenges in the comparison of activity. These phenomena can be ascribed to the complex reaction mechanism and the difficulty in the product analysis. Further studies in this area are still necessary.

Pure Nb_2_O_5_, N–Nb_2_O_5_, and composited Nb_2_O_5_ photocatalysts were also applied in the removal of toxic Cr(VI) species (Table 1, Nos. 156–163).^[^ ^77^, ^99^, ^117^ ^]^ Unlike the degradation of organic pollutants, the Cr(VI) species are reduced by the photogenerated electrons (Figure 7, path C). Besides, the utilization of Nb_2_O_5_‐based photocatalysts was also reported in the inactivation of bacteria (Table 1, Nos. 164–166). The *Staphylococcus aureus* and *Escherichia coli* were inactivated by •O_2_^−^ species that were generated from the reaction between O_2_ and excited electrons on the Nb_2_O_5_ surface (Figure 7, path A).^[^ ^77^ ^]^ Considering the nontoxic demand for drugs, these results imply the potential application of Nb_2_O_5_‐based photocatalysts in the medical field.

### 4.2 Photocatalytic H_2_ and O_2_ Evolution

Photocatalytic hydrogen evolution is the potential process to produce H_2_ as a clean energy carrier.^[^ ^5^, ^204^ ^]^ Generally, the excited electrons in semiconductor photocatalysts are utilized for the reduction of H^+^ ions or H_2_O to H_2_.^[^ ^5b^ ^]^ However, the reaction rate over Nb_2_O_5_ is limited by several factors, including i) the high recombination efficiency of charge carriers, ii) the low reduction rate of catalytic protons to H_2_, iii) the insufficient absorption capacity of visible light, and so on. Thus, some strategies have been developed to enhance the photocatalytic performance.^[^ ^5b^ ^]^ For instance, sacrificial agents, like triethanolamine (TEOA), methanol, lactic acid, Na_2_S, and Na_2_SO_3_, were introduced into the reaction mixture to consume the holes on Nb_2_O_5_‐based photocatalysts while the remaining electrons were still utilized for the production of H_2_ (**Table** **2**, Nos. 1–32).^[^ ^103^, ^205^ ^]^ Modified Nb_2_O_5_ with platinum as a cocatalyst was introduced to promote the reduction of protons to H_2_ (Table 2, Nos. 1–5). Similarly, Nb_2_O_5_ can be modified with Au and sulfide to enhance the hydrogen evolution rate (Table 2, Nos. 24, 28–29, 31). Meanwhile, the obtained catalyst is active under visible light irradiation (Table 2, No. 4).

**Table 2:** Recent advances in the photocatalytic H_2_ and O_2_ evolution over Nb_2_O_5_‐based photocatalysts

No.	Catalysts	Products	Sacrificial agents	Light sources	Reaction temperature [°C]	Reaction rate [µmol g^−1^ h^−1^]	AQY^a)^ [%]	Refs.
1	Pt/H‐Nb_2_O_5_ nanorods	H_2_	Methanol	500 W Hg lamp	n.m.^b)^	≈1820	n.m.	^[^ ^205b^ ^]^
2	Pt/TT‐Nb_2_O_5_ nanowires	H_2_	Methanol	300 W Xe lamp	25	680	4.7	^[^ ^40a^ ^]^
3	Pt/TT‐Nb_2_O_5_ nanowires	H_2_	Methanol	300 W Xe lamp (*λ* > 300 nm)	n.m.	≈780	4.6	^[^ ^40b^ ^]^
4	Pt/N‐HNb_3_O_8_ nanosheets	H_2_	Methanol	300 W Xe lamp (*λ* > 420 nm)	n.m.	≈1200	1.69	^[^ ^50f^ ^]^
5	Pt/Nb_2_O_5_	H_2_	Methanol	400 W Hg lamp	20	12 350	n.m.	^[^ ^20^ ^]^
6	CuO/Nb_2_O_5−_ *_x_*	H_2_	Methanol	300 W white light	50	1405	n.m.	^[^ ^69^ ^]^
7	Nb_2_O_5_ nanoparticles	H_2_	Methanol	300 W Hg lamp	25–27	191	n.m.	^[^ ^208^ ^]^
8	Pt/C–Nb_2_O_5_	H_2_	Methanol	300 W Xe lamp (*λ* > 420 nm)	n.m.	≈39	n.m.	^[^ ^82b^ ^]^
9	Pt/N–Nb_2_O_5_	H_2_	Methanol	150 W Xe lamp (*λ* > 400 nm)	25	154	n.m.	^[^ ^19^ ^]^
10	Pt/N–Nb_2_O_5_	H_2_	Methanol	400 W Hg lamp	r.t.^c)^	3010	n.m.	^[^ ^84^ ^]^
11	N–Nb_2_O_5_/r‐GO	H_2_	Methanol	Sunlight	n.m.	5370	4.5	^[^ ^83a^ ^]^
12	Carbonaceous Nb_2_O_5_	H_2_	Methanol	500 W Xe lamp	n.m.	2	n.m.	^[^ ^209^ ^]^
13	Pt/Nb_2_O_5_	H_2_	Methanol	400 W halide lamp	43	4647	n.m.	^[^ ^95b^ ^]^
14	Pt/Nb_2_O_5_	H_2_	Methanol	150 W solar simulator	r.t.	≈25	1.06	^[^ ^210^ ^]^
15	Pt/Nb_2_O_5_	H_2_	Methanol	165 W Hg lamp	10	9790	n.m.	^[^ ^211^ ^]^
16	NiO QDs/Nb_2_O_5_	H_2_	Methanol	300 W Xe lamp	n.m.	124	n.m.	^[^ ^212^ ^]^
17	Pt/Nb_2_O_5_/TiO_2_	H_2_	Methanol	200 W Xe lamp (320–780 nm)	n.m.	1800	n.m.	^[^ ^213^ ^]^
18	Er–Y_3_Al_5_O_12_@ Nb_2_O_5_/Pt/In_2_O_3_	H_2_	Methanol	300 W Xe lamp (420–800 nm)	25	≈100	n.m.	^[^ ^214^ ^]^
19	Nb_2_O_5_/MoS_2_/graphene	H_2_	Methanol	Visible light	r.t.	136 800	n.m.	^[^ ^215^ ^]^
20	Nb_2_O_5_/C/Nb_2_C	H_2_	Methanol	200 W Hg lamp	25	≈8	0.11	^[^ ^97d^ ^]^
21	Pt/Nb_2_O_5_–r‐GO	H_2_	Methanol	150 W Xe lamp (*λ* > 400 nm)	25	≈882	13	^[^ ^216^ ^]^
22	Pt/Nb_2_O_5_–N‐doped graphene	H_2_	Methanol	150 W Xe lamp (*λ* > 400 nm)	r.t.	≈24	n.m.	^[^ ^97e^ ^]^
23	Pt/Nb_2_O_5_	H_2_	Methanol	400 W Hg lamp	n.m.	1120	>6	^[^ ^70^ ^]^
24	Au/Nb_2_O_5_	H_2_	Methanol	500 W Xe lamp	n.m.	≈11	n.m.	^[^ ^166^ ^]^
25	Pt/C‐modified Nb_2_O_5_	H_2_	Methanol	300 W Xe lamp (*λ* > 420 nm)	n.m.	7	n.m.	^[^ ^171^ ^]^
26	Pt/g‐C_3_N_4_/Nb_2_O_5_	H_2_	TEOA^d)^	300 W Xe lamp (*λ* > 400 nm)	<6	1710	n.m.	^[^ ^99g^ ^]^
27	Pt/g‐C_3_N_4_/Nb_2_O_5_	H_2_	TEOA	1000 W Xe lamp	n.m.	110 000	n.m.	^[^ ^205d^ ^]^
28	Pt/Nb_2_O_5_/ZnIn_2_S_4_	H_2_	TEOA	300 W Xe lamp	5	6026	3.75	^[^ ^217^ ^]^
29	Nb_2_O_5_–SnS_2_–CdS	H_2_	Lactic acid	300 W Xe lamp	r.t.	≈3600	0.65	^[^ ^205a^ ^]^
30	Pt/Nb_2_O_5_	H_2_	Na_2_SO_3_	300 W Xe lamp (*λ* > 420 nm)	n.m.	130	n.m.	^[^ ^205c^ ^]^
31	CdS/Nb_2_O_5_/N‐doped graphene	H_2_	Na_2_S and Na_2_SO_3_	150 W Xe lamp (*λ* > 400 nm)	25	≈96	1.5	^[^ ^103b^ ^]^
32	TT‐Nb_2_O_5_ nanowires	H_2_	Na_2_S and Na_2_SO_3_	500 W Xe lamp (*λ* > 420nm)	n.m.	≈244	n.m.	^[^ ^183^ ^]^
33	Pt/TT‐Nb_2_O_5_ nanowires	O_2_	AgNO_3_	300 W Xe lamp	25	70	n.m.	^[^ ^40a^ ^]^
34	TT‐Nb_2_O_5_ nanowires	O_2_	AgNO_3_	300 W Xe lamp (*λ* > 300 nm)	n.m.	≈620	n.m.	^[^ ^40b^ ^]^

^a)^ Apparent quantum yield

^b)^ Not mentioned

^c)^ Room temperature

^d)^ Triethanolamine.

Furthermore, the Nb_2_O_5_ and Pt/Nb_2_O_5_ were applied in the oxidation of water to O_2_ (Table 2, Nos. 33–34). In this process, AgNO_3_ acted as the sacrificial agent, which was reduced by the photogenerated electrons on Nb_2_O_5_‐based photocatalysts. Especially, the apparent quantum yield (AQY) was mentioned in these processes (Table 2, Nos. 2–4), which is defined by the number of the reacted electrons to the number of incident photons.^[^ ^206^ ^]^ The AQY can be a benchmark for comparison of efficiency in different photocatalytic systems. Recently, the AQY in the photocatalytic water splitting to H_2_ and O_2_ was up to ≈96% on Al–SrTiO_3_ under 360 nm light irradiation.^[^ ^207^ ^]^ This result is much higher than that reported on Nb_2_O_5_‐based catalysts (Table 2, Nos. 2–4). Hence, the challenge and opportunity are still present in further improving the activity of Nb_2_O_5_‐based photocatalysts.

### 4.3 Photoreduction of CO_2_

CO_2_ as a carbonaceous resource can be applied in the production of chemicals and fuels.^[^ ^218^ ^]^ For instance, CO_2_ can be reduced to one‐carbon (C_1_) molecules, like CO, HCOOH, HCHO, CH_3_OH, and CH_4_, and C_2+_ products.^[^ ^218^ ^]^ There are two typical reaction modes for photocatalytic reduction of CO_2_: solid–liquid interface reaction mode (mode I) and solid–vapor interface reaction mode (mode II).^[^ ^219^ ^]^ In the first mode, the photocatalysts were introduced into an aqueous solution. Dissolved CO_2_ in water can be reduced on the solid–liquid interface. For another one, CO_2_ molecules were directly reduced on the solid‐photocatalysts surface. Especially, two modes were both reported with the utilization of Nb_2_O_5_‐based photocatalysts.^[^ ^9^, ^220^ ^]^ The CO, HCOOH, CH_3_OH, CH_4_, and CH_3_COOH were observed in these reduction process (**Table** **3**).^[^ ^9^, ^220^ ^]^ Because the dissolved CO_2_ in water is limited in mode I that was widely reported, sacrificial agents, such as triethylamine, were added to improve the solubility of CO_2_ in water and consumed the excited holes. Besides, the photoreduction of CO_2_ can be occurred on amorphous Nb_2_O_5_ without any additives (Table 3, No. 3). Some possible reaction pathways were proposed as following Equations (1)–(8)^[^ ^9^, ^219^ ^]^

(1) $$CO_{2}+2H^{+}+2e^{-}\to \mathrm{HCOOH}$$

(2) $$CO_{2}+2H^{+}+2e^{-}\to \mathrm{CO}+H_{2}O$$

(3) $$CO_{2}+4H^{+}+4e^{-}\to \mathrm{HCHO}\;+\;H_{2}O$$

(4) $$CO_{2}+6H^{+}+6e^{-}\to CH_{3}\mathrm{OH}\;+\;H_{2}O$$

(5) $$CO_{2}+8H^{+}+8e^{-}\to CH_{4}+2H_{2}O$$

(6) $$CO_{2}+H^{+}+e^{-}\to •\mathrm{COOH}$$

(7) 2•COOH→HCOOCOOH

(8) $$\mathrm{HCOOCOOH}\;\;+\;\;H^{+}+e^{-}\to CH_{3}\mathrm{CHOOH}$$

**Table 3:** Recent advances in the photocatalytic reduction of CO_2_ over Nb_2_O_5_‐based photocatalysts

No.	Catalysts	Substrates	Main product	Light sources	Reaction temperature [°C]	Reaction rate [µmol g^−1^ h^−1^]	Refs.
1	In_2_O_3−_ *_x_*(OH)*_y_*/Nb_2_O_5_ nanorods	CO_2_ and H_2_	CO	300 W Xe lamp	60	1400	^[^ ^220a^ ^]^
2	HNb_3_O_8_ nanobelts	CO_2_ and H_2_O	CH_4_	350 W Xe lamp	45	3.58	^[^ ^220b^ ^]^
3	Amorphous Nb_2_O_5_	CO_2_ and H_2_O	CH_3_COOH	UV light	n.m.^a)^	≈1.35	^[^ ^9b^ ^]^
4	SiO_2_–HNb_3_O_8_	CO_2_ and H_2_O	CH_4_	350 W Xe lamp	60	2.90	^[^ ^220c^ ^]^

^a)^ Not mentioned.

It is very important to underline some critical, analytical, and mechanistic aspects in the photocatalytic conversion of CO_2_. Over the past decade, it is known that carbon residues can be involved in photocatalytic water activation and CO_2_ reduction.^[^ ^221^ ^]^ This is particularly relevant for the correct evaluation of the rates of artificial photosynthesis using photocatalysts synthesized with carbon‐containing precursors. For this reason, it has become more and more relevant to the use of ^13^CO_2_ to prove the mechanism of CO_2_ reduction. In fact, the reaction products, often in trace levels, can derive also from light‐induced desorption or reaction of carbonaceous impurities or residues from the synthesis in organic media that are not fully removed even by calcination. For instance, the CH_4_ can be observed from the catalysts under light irradiation without CO_2_.^[^ ^220c^ ^]^ After eliminating the effects of carbon residues, the experimental results are conducive to reveal the process of photocatalytic reduction of CO_2_. The photocatalytic efficiency can be evaluated by the AQY, which was not mentioned in these processes (Table 3).^[^ ^9^, ^220^ ^]^ Besides, the selectivity of products is also important for catalytic performance. The C_1_ products from CO_2_ are important chemical intermediates and fuels.^[^ ^218^ ^]^ High selectivity (>99%) of CO and CH_4_ has been obtained, respectively (Table 3, Nos. 1–2). Although other acid products were observed in pure Nb_2_O_5_,^[^ ^9d^ ^]^ the selectivity of HCOOH (35%) was competitive with that of CH_3_COOH (42%; Table 3, No.3). The formation of CH_3_COOH involved the C—C coupling reaction of •COOH radicals (Equations (6)–(8)).^[^ ^9a^ ^]^ Unfortunately, the uncontrollable activity of •COOH radicals leads to the simultaneous generation of CH_3_COOH and HCOOH.^[^ ^115^ ^]^ Besides, syngas that is vital in Fischer–Tropsch synthesis can be directly obtained from the reduction of CO_2_ and H_2_ evolution in photocatalysis.^[^ ^222^ ^]^ To date, such processes are yet to be recognized over Nb_2_O_5_‐based photocatalysts.

### 4.4 Selective Transformation of Organic Molecules

Amines, aldehydes, and ketones are important organic intermediates for medicines and polymers.^[^ ^223^ ^]^ The VB maximum of Nb_2_O_5_ is up to ≈+2.50 V versus NHE (normal hydrogen electrode), implying its potential application in the oxidation and succedent transformation of organic molecules.^[^ ^9a^ ^]^ The amines, alcohols, propene, cyclohexane, toluene, and ethylbenzene were selective oxidation to corresponding imines, aliphatic aldehydes, ketones, benzaldehyde, and acetophenone (**Table** **4**). Similar to the photocatalytic reduction of CO_2_ (Section 4.3), there were also two typical reaction modes in the selective transformation of organic molecules. In the first mode, O_2_, solid photocatalysts, pure organic liquid, or the substrate dissolved in the solvent, like benzene and acetonitrile, were present in the system (Table 4, Nos. 1–16). For another one, the mixture of O_2_, substrate, and solid photocatalysts were introduced into the reactor (Table 4, No. 17). The reaction rate of benzylamine observed on Nb_2_O_5_ was higher than that of TiO_2_.^[^ ^9c^ ^]^ Meanwhile, the selectivity of *N*‐benzylidene benzylamine on Nb_2_O_5_ is up to 98%.^[^ ^9c^ ^]^ Besides, the selectivity of partial oxidation products was up to 97% after the deposition of Nb_2_O_5_ on the TiO_2_ surface under UV light irradiation.^[^ ^97a^ ^]^ This may be attributed to that the amounts of photogenerated O_3_^−^ species over the catalyst drastically decreased, which were estimated by electron spin resonance spectroscopy.^[^ ^97a^ ^]^ Interestingly, primary alcohols oxidized to aldehydes without the generation of acid on Nb_2_O_5_ under visible light irradiation.^[^ ^224^ ^]^ A detailed relationship between product selectivity and structure of Nb_2_O_5_‐based photocatalysts is summarized in the next section.

**Table 4:** Recent advances in the selective photooxidation of organic molecules over Nb_2_O_5_‐based photocatalysts

No.	Catalysts	Substrates	Main products	Light sources	Reaction temperature [°C]	Reaction rate [µmol g^−1^ h^−1^]^a)^	AQY [%]	Refs.
1	HNb_3_O_8_ nanosheets	Amines	Imines	300 W Xe lamp (*λ* > 420 nm)	25	≈1979	6.57	^[^ ^12c^ ^]^
2	Nb_2_O_5_	Amines	Imines	500 W Hg lamp	r.t.^b)^	1298	≈14	^[^ ^9c^ ^]^
3	Nb_2_O_5_@NiFe‐MMO	Benzyl‐amine	Imine	300 W Xe lamp	30	≈18281	n.m.^c)^	^[^ ^226^ ^]^
4	Nb_2_O_5_/ZnMgAl‐LDH	Anilines	Azoxy‐benzenes	50 W violet light LED	r.t.	≈1979	n.m.	^[^ ^227^ ^]^
5	Nb_2_O_5_	1‐pentanol	Pentanal	500 W Hg lamp	50	≈1.28	n.m.	^[^ ^228^ ^]^
6	HNb_3_O_8_ nanosheets	Benzylic alcohols	Benz‐aldehyde	300 W Xe lamp (*λ* > 400 nm)	25	≈1969	n.m.	^[^ ^229^ ^]^
7	Nb_2_O_5_	Alcohols	Aldehydes and ketones	500 W Hg lamp (*λ* > 390 nm)	50	≈8.97	≈5.2	^[^ ^230^ ^]^
8	Nb_2_O_5_	Alcohols	Aldehydes and ketones	500 W Hg lamp (*λ* > 390 nm)	r.t.	≈619	n.m.	^[^ ^224^ ^]^
9	Nb_2_O_5_	HMF	DFF	300 W Xe lamp	30	≈333	n.m.	^[^ ^9e^ ^]^
10	Nb_2_O_5_/TiO_2_	1‐pentanol	Pentanal	500 W Hg lamp	r.t.	≈8660	n.m.	^[^ ^97a^ ^]^
11	Nb_2_O_5_/TiO_2_	Alcohols	Aldehydes and ketones	500 W Hg lamp	r.t.	≈48748	n.m.	^[^ ^231^ ^]^
12	Nb_2_O_5_/TiO_2_	Aryl alcohols	Aldehydes and ketones	200 W Xe lamp	n.m.	17 600	n.m.	^[^ ^213^ ^]^
13	Nb_2_O_5_/SiO_2_	Ethanol	Acet‐aldehyde	500 W Hg lamp (*λ* > 320 nm)	37	≈107	n.m.	^[^ ^232^ ^]^
14	Nb_2_O_5_	CH^d)^ and EB^e)^	Aldehydes and ketones	500 W Hg lamp (*λ* > 390 nm)	r.t.	≈120	n.m.	^[^ ^9d^ ^]^
15	Nb_2_O_5_	Toluene	Benz‐aldehyde	200 W Hg‐Xe lamp (*λ* > 390 nm)	20	≈80	≈11	^[^ ^18^ ^]^
16	N–Nb_2_O_5_	Toluene	Benz‐aldehyde	6 W LED	40	≈28	n.m.	^[^ ^12b^ ^]^
17	Nb_2_O_5_/SiO_2_	Propene	Aldehydes	500 W Xe lamp (*λ* > 290 nm)	r.t.	≈13	n.m.	^[^ ^233^ ^]^
18	Pd/HNb_3_O_8_ nanosheets	Aryl nitro‐compound	Aniline	300 W Xe lamp (*λ* > 320 nm)	25	≈2168	n.m.	^[^ ^50c^ ^]^
19	Nb_2_O_5_ nanosheet	Plastics	CH_3_COOH	300 W Xe lamp	25	≈0.79	n.m.	^[^ ^9a^ ^]^
20	Au/Nb_2_O_5_	Methanol	DMM^f)^	UV light	25	≈2.64	n.m.	^[^ ^225^ ^]^
21	Nb_2_O_5_/ZnIn_2_S_4_	HMF^g)^ and H_2_O	DFF^h)^ and H_2_	Simulated solar light	30	≈429	n.m.	^[^ ^217^ ^]^

^a)^ Productivity of the main product

^b)^ Room temperature

^c)^ Not mentioned

^d)^ Cyclohexane

^e)^ Ethylbenzene

^f)^ Dimethoxymethane

^g)^ 5‐Hydroxymethylfurfural

^h)^ 2,5‐Diformylfuran.

In addition, Pd/HNb_3_O_8_ nanosheets were efficient in the reduction of aryl nitro‐compounds to aniline (Table 4, No. 18).^[^ ^50c^ ^]^ Moreover, Nb_2_O_5_ catalysts were also applied in the photocatalytic coupling reaction. Xie's group demonstrated that the polyethylene was completely photodegraded on Nb_2_O_5_ nanosheets while generated CO_2_ was further reduced to CH_3_COOH (Table 4, No. 19).^[^ ^9a^ ^]^ Possible reaction mechanism was provided, in which CO_2_ was reduced to •COOH radicals, HCOOCOOH, and CH_3_COOH (Section 4.3, Equations (6)–(8)).^[^ ^9a^ ^]^ The transformation of waste plastics to chemicals and fuel can be realized by this process. Although the yield of CH_3_COOH is limited, further designs in photocatalysts are possible to enhance its activity. In addition, the acid sites on Nb_2_O_5_ play an important role in the coupling reaction. For instance, dimethoxymethane molecules were generated in the photooxidation methanol, indicating the coupling formaldehyde and methanol catalyzed by the BAS of Au/Nb_2_O_5_ (Table 4, No. 20).^[^ ^225^ ^]^ The other example reported by Lei's group demonstrated that 2,5‐diformylfuran (DFF) and H_2_ are produced from 5‐hydroxymethylfurfural (HMF) and H_2_O on Nb_2_O_5_/ZnIn_2_S_4_, in which HMF acted as a sacrificial agent to consume the holes to improve the evolution of H_2_ with the formation of DFF (Table 4, No. 21).^[^ ^217^ ^]^ This strategy is available to improve the efficiency of photogenerated holes and electrons simultaneously.^[^ ^7a^ ^]^

## 5 The Structure–Activity Relationship

### 5.1 The Role of Size and Crystalline Phases

**Figure 8:**
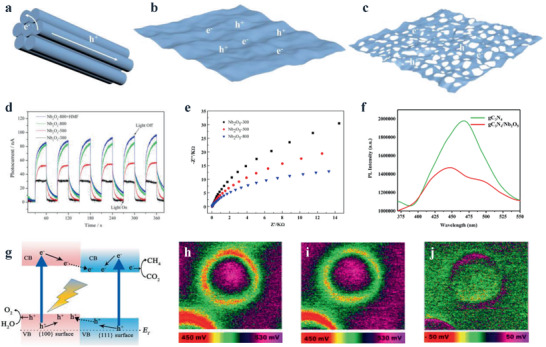
a–c) The distribution of charge carriers on different Nb_2_O_5_ catalysts. d) The photocurrent response,^[^ ^9e^ ^]^ e) electrochemical impedance spectroscopy,^[^ ^9e^ ^]^ and f) photoluminescence spectroscopy of Nb_2_O_5_‐based catalysts.^[^ ^105b^ ^]^ g) The charge migration on CeO_2_.^[^ ^240^ ^]^ SPVM image of Au/TiO_2_ h) under dark and i) under 532 nm illumination, respectively.^[^ ^238a^ ^]^ j) The different spectrum from (i) and (h).^[^ ^238a^ ^]^ d,e) Reproduced with permission.^[^ ^9e^ ^]^ Copyright 2017, American Chemical Society. f) Reproduced with permission.^[^ ^105b^ ^]^ Copyright 2019, American Chemical Society. g) Reproduced with permission.^[^ ^240^ ^]^ Copyright 2015, American Chemical Society. h‐j) Reproduced with permission.^[^ ^239^ ^]^ Copyright 2017, American Chemical Society.

Previously, Nb_2_O_5_‐based catalysts with high SSA can be efficient photocatalysts (**Figure** **8**a–c).^[^ ^70^ ^]^ In the degradation process of trichloroethylene, T‐Nb_2_O_5_ nanotubes showed higher activity than that of layered K_4_Nb_6_O_17_, which were ascribed to their higher crystallinity and specific surface area.^[^ ^23c^ ^]^ After that, Zhang's group reported that H‐Nb_2_O_5_ nanorods exhibited higher photocurrent density than that of commercial counterpart, due to the positive effect of high SSA on the separation of photogenerated carriers.^[^ ^205b^ ^]^ Besides, the thickness of Nb_2_O_5_‐based catalysts may play an important role in photocatalysis. Tsang's group reported that a higher photocatalytic H_2_ evolution rate was observed with smaller numbers of the layer on Nb_2_O_5_‐based nanosheets.^[^ ^12^, ^43^ ^]^ In Yu's results, the photocatalytic H_2_ evolution rate was enhanced with a decrease in the wall thickness of porous Nb_2_O_5_.^[^ ^20^ ^]^ These results imply a positive role of the thin‐walled structure of Nb_2_O_5_ in the separation of charge carriers.

Although the crystal faces of Nb_2_O_5_, like (010), were revealed by the high‐resolution transmission electron microscopy (HRTEM), the role of these structures is rarely reported in photocatalysis.^[^ ^33^, ^234^ ^]^ Previously, Kudo's group afforded a possible explanation.^[^ ^40b^ ^]^ In the photocatalytic H_2_ evolution, higher activity of TT‐Nb_2_O_5_ nanowires was observed than that of bulk counterpart in their results.^[^ ^40b^ ^]^ Meanwhile, the Pt particles were selectively distributed on the short‐axis plane of the TT‐Nb_2_O_5_ nanowires in the photodeposition process. These results suggested that the photogenerated electrons moved along the nanowire growth direction while holes migrated to the nanowire sidewall.^[^ ^40b^ ^]^ Although some Pt particles were also observed on other facets, Kudo's group described the mobility difference of charge carriers in the crystal growth direction. After that, the driving force can be ascribed to the formation of a built‐in electric field between different facets, which is instructive to the separation of e^−^ and h^+^.^[^ ^235^ ^]^ In addition, Tsang's group reported that {001} facet on Nb_2_O_5_ nanorods was active for photodegradation of methylene blue.^[^ ^33a^ ^]^ This can be attributed to the strong Lewis acidity of Nb_2_O_5_ nanorods, which is summarized in Section 5.3.^[^ ^33a^ ^]^

Previously, TT‐Nb_2_O_5_ exhibited a higher SSA and reactivity than those of T‐Nb_2_O_5_ and H‐Nb_2_O_5_.^[^ ^9^, ^70^, ^135^ ^]^ Whereas, some research groups found that T‐Nb_2_O_5_ and H‐Nb_2_O_5_ showed higher activity than that of TT‐Nb_2_O_5_ in photodegradation of methylene blue and selective oxidation of 5‐hydroxymethylfurfural (Table 4, No. 9).^[^ ^9^, ^135^ ^]^ For instance, the T‐Nb_2_O_5_ obtained by calcination at 800 °C showed the highest photocurrent density than those of counterparts treated at 300 and 500 °C.^[^ ^9e^ ^]^ Except for the SSA, these results indicated other factors might play an important role in photocatalysis. Previously, the formation energy of oxygen vacancy is changed on different crystalline phases of metal oxide.^[^ ^236^ ^]^ As revealed by the results of X‐ray photoelectron spectra (XPS), a high concentration of oxygen vacancies was observed on H‐Nb_2_O_5_.^[^ ^135^ ^]^ These results suggest the positive effect of oxygen vacancy induced by phase transformation in photocatalysis. The detailed discussion of the role of oxygen vacancy is shown in Section 5.2.

As mentioned above, the photochemical characterizations of catalysts are necessary to reveal the distribution and migration of charge carriers. The photocurrent response, electrochemical impedance spectroscopy (EIS), and photoluminescence spectroscopy (PL) are developed to verify the separation of electrons and holes (Figure 8d–f). Besides, the theoretic calculation was utilized to study the transfer process of excited holes and electrons on metal oxide (Figure 8g).^[^ ^237^, ^240^ ^]^ Recently, the charge carriers can be directly detected by surface photovoltage microscopy (SPVM).^[^ ^238^ ^]^ As shown in Figure 8h–j, a circular ring was observed on the differential spectrum of surface photovoltage over Au/TiO_2_, which is corresponding to the accumulation of excited holes in the interface (Au—O—Ti) under light irradiation.^[^ ^239^ ^]^ In principle, these characterization techniques are universal and conducive to the profound understanding of the spatial distribution of charge carriers on Nb_2_O_5_.

### 5.2 The Role of Unsaturated Nb Sites and Oxygen Vacancies

The oxygen vacancies of Nb_2_O_5_ play important roles in the absorption and activation of the substrate.^[^ ^229^, ^241^ ^]^ Previously, the unsaturated Nb sites were observed with the formation of oxygen vacancies, which were revealed by the results of electron paramagnetic resonance (EPR) and XPS. ^[^ ^229^, ^241^ ^]^ The EPR signal at 2.003 is assigned to the oxygen vacancy.^[^ ^229^ ^]^ After the adsorption of BA on HNb_3_O_8_ nanosheets, the intensity of the signal at 2.003 was weakened. Meanwhile, the characteristic O—H and C—O bands of BA were shifted to the lower wavenumber in Fourier transform infrared spectra (FT‐IR), indicating that the BA molecules were adsorbed on unsaturated Nb sites by the formation of C—O—Nb complex.^[^ ^229^ ^]^ As a result, the optical absorption edge of BA/HNb_3_O_8_ nanosheets was extended to visible light, indicating that this structure was beneficial for the migration of charge carriers.^[^ ^229^ ^]^

Besides, the unsaturated Nb sites and oxygen vacancies are conducive to the separation of charge carriers.^[^ ^50c^ ^]^ The HNb_3_O_8_ nanosheets with abundant oxygen vacancies exhibited a faster reaction rate and higher separation efficiency of charge carriers than those of urchin‐like Nb_2_O_5_ and Nb_2_O_5_ nanorods, as shown by the results of EIS, photocurrent response.^[^ ^12c^ ^]^ Meanwhile, PL analysis and time‐resolved fluorescence (TRF) results suggested that the oxygen vacancies were conducive to the separation of photogenerated holes and electrons.^[^ ^77^ ^]^ In addition, the optical absorption ability of catalysts can be affected by the unsaturated Nb sites and oxygen vacancies.^[^ ^17^, ^242^ ^]^ The HNb_3_O_8_ nanosheets exhibited a wide bandgap (≈3.4 eV), corresponding to the absorption spectrum within 370 nm.^[^ ^90^ ^]^ With the formation of oxygen vacancies, the bandgap of HNb_3_O_8_ nanosheets was narrowed.^[^ ^50f^ ^]^ Moreover, new energy states with a small bandgap (<0.5 eV) were observed with the increasing concentration of surface unsaturated Nb sites and oxygen vacancies.^[^ ^17c^ ^]^ This electronic structure can be utilized for the absorption of the full solar spectrum (250–2500 nm).^[^ ^17c^ ^]^ Thus, heat sourced from the absorption of infrared light over HNb_3_O_8_ nanosheets contributed to enhancing reaction rate in kinetics.^[^ ^17c^ ^]^ The inert C—H bonds of hydrocarbons can be activated by the holes generated at the valence band under UV light irradiation in the thermodynamics.^[^ ^9a^ ^]^ These results shed light on the balance of electronic structure and concentration of oxygen vacancies for photocatalyst design and preparation.

Especially, the unsaturated Nb sites and oxygen vacancies can be formed without any sacrificial agents under UV light irradiation, indicating that light irradiation can be utilized for the modification of catalyst.^[^ ^50c^ ^]^ Meanwhile, the concentration of oxygen vacancies was not constant and changed under irradiation.^[^ ^50c^ ^]^ To reveal the structure of Nb_2_O_5_ in situ under light irradiation, theoretical calculations in oxygen‐vacancy formation energy on different facets and crystalline phases are necessary for the profound understanding of these phenomena.^[^ ^9e^ ^]^

### 5.3 The Role of LAS, BAS, and Acidity

There are consecutive tandem steps involved in photocatalytic processes: 1) light‐harvesting on photocatalysts, 2) separation and migration of photogenerated holes and electrons, and 3) succedent surface redox reactions.^[^ ^243^ ^]^ Particularly, the surface redox reactions and selectivity of products are related to the acid–base properties of photocatalysts.^[^ ^243^ ^]^ Previously, propene was oxidized to propanal and acetaldehyde on Nb_2_O_5_/SiO_2_ photocatalysts under light irradiation.^[^ ^233^ ^]^ The selectivity of propanal was much different on the Nb_2_O_5_/SiO_2_ catalysts.^[^ ^105a^ ^]^ As revealed by results of x‐ray absorption near edge structure (XANES) and extended x‐ray absorption fine structure (EXAFS), tetrahedral NbO_4_ and octahedral NbO_6_ units were present on 0.66 and 4.6 wt% Nb_2_O_5_/SiO_2_, respectively.^[^ ^233^ ^]^ Notably, the NbO_6_ octahedra acted as the BAS.^[^ ^14a^ ^]^ The LAS can be observed with the formation of NbO_5_ and NbO_4_ polyhedron.^[^ ^15^ ^]^ The roles of acid sites are possibly revealed by these results. However, other products, like acetone and acrolein, were also observed, leading to the difficulty in the understanding of the relationship between BAS, LAS, and photooxidation processes.^[^ ^105a^ ^]^

**Figure 9:**
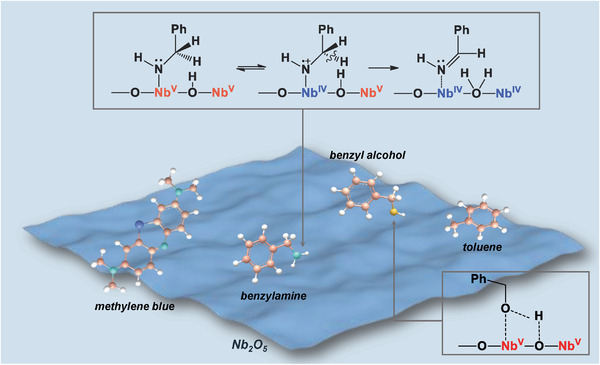
The schematic illustration of methylene blue, benzylamine, benzyl alcohol, and toluene adsorbed on Nb_2_O_5_. Adapted with permission.^[^ ^230^ ^]^ Copyright 2009, American Chemical Society. Adapted with permission.^[^ ^248^ ^]^ Copyright 2012, American Chemical Society.

After that, the unique acidity of Nb_2_O_5_ was found to be instructive to the adsorption–activation process in the photooxidation reaction.^[^ ^14^, ^244^ ^]^ The deep oxidation products of alcohols were observed with increasing Lewis acidity of Nb_2_O_5_, which may be ascribed to the strong adsorption of aldehydes intermediates on LAS.^[^ ^228^ ^]^ Besides, the Nb_2_O_5_–amide surface complex was generated by the adsorption of amines on Nb_2_O_5_.^[^ ^245^ ^]^ This complex can be excited by light (*λ* > 390 nm) with lower energy than that of Nb_2_O_5_.^[^ ^245^ ^]^ Meanwhile, the yield and selectivity of benzylamine to *N*‐benzylidene benzylamine over commercial Nb_2_O_5_ were higher than those over TiO_2_ under visible light irradiation.^[^ ^9c^ ^]^ This is mainly due to the activation of amines adsorbed on Nb_2_O_5_ via the ligand‐to‐metal charge transfer (LMCT) transition (**Figure** **9**).^[^ ^245^ ^]^ The electrons transferred from the N 2p orbitals of amides to Nb 4d orbitals of Nb_2_O_5_.^[^ ^244^ ^]^ Then, the *α*‐C—H bonds were activated to form the imines.^[^ ^245^ ^]^ This direct excitation of amines is beneficial to improve the selectivity of the product by inhibiting the generation of other oxygen‐containing species, such as ozonide anion radicals (O^3−^) and hydroxyl radicals (HO•).^[^ ^246^ ^]^ Thus, the undesired deep oxidation was restricted under visible light irradiation.^[^ ^246^ ^]^ Similarly, alcohols were selectively oxidized to corresponding aldehydes without further oxidation to carboxylic acids.^[^ ^224^ ^]^ When the surface isolated —OH groups on Nb_2_O_5_ were partly removed by a vacuum heat treatment, inert aromatic hydrocarbons with relatively large ionization energy also can be transformed to aldehydes under visible light irradiation (Figure 9).^[^ ^18^ ^]^ However, the active sites on Nb_2_O_5_ are still unclear for the activation of the inert sp^3^ C—H bond on toluene.^[^ ^12^, ^18^ ^]^ Besides, Tsang's group proposed the adsorption–activation process of dyes on Nb_2_O_5_.^[^ ^33a^ ^]^ In their results, Nb_2_O_5_ nanorods exhibited higher activity than that of ZnO in the photocatalytic degradation of methylene blue.^[^ ^33a^ ^]^ After the introduction of a radical scavenger, the comparable activity of Nb_2_O_5_ nanorods was observed, indicating that hydroxyl radicals (HO•) played a marginal role in the reaction.^[^ ^33a^ ^]^ As revealed by FT‐IR results, the number of methyl blue molecules adsorbed on Nb_2_O_5_ nanorods was higher than that on ZnO, implying that the strong Lewis acidity of Nb_2_O_5_ nanorods was beneficial for the adsorption–activation process of methylene blue.^[^ ^33a^ ^]^

In addition, the acidity of Nb_2_O_5_ is also associated with the selectivity of products in the photoreduction reaction. The photoreduction of CO_2_ to CH_4_ was dominated on HNb_3_O_8_ nanosheets and SiO_2_‐pillared HNb_3_O_8_ that mainly exposed the BAS.^[^ ^15^, ^220^ ^]^ The yield of CH_4_ reached 2.9 µmol g_cat._^−1^ h^−1^ over SiO_2_‐pillared HNb_3_O_8_, which is much higher than that over HNb_3_O_8_ (0.47 µmol g_cat._^−1^ h^−1^), implying the promoting effects of BAS derived from the dispersed HNb_3_O_8_ on SiO_2_.^[^ ^220c^ ^]^ Meanwhile, Ribeiro's group found a quite different tendency that improving the number of acid sites on Nb_2_O_5_ can promote the photoreduction of CO_2_ to CO, HCOOH, and CH_3_COOH.^[^ ^9b^ ^]^ As shown in Equations (1)–(8) (Section 4.3), protons are vital in the reduction of CO_2_ to CH_4_. However, the number of acid sites on different Nb_2_O_5_ samples was measured by the ionic‐exchange and titration approach, leading to unclear amounts of BASs on the surface of catalysts.^[^ ^9b^ ^]^ Meanwhile, the activation of CO_2_ is related to the local structure of Nb—O—Nb.^[^ ^247^ ^]^ These results lead to an ambiguous understanding of the photoreduction of CO_2_. Hence, further studies are still necessary to get insight into the relationship between the surface —OH groups, NbO*_x_* units, BAS, LAS, and product selectivity in the photoreduction of CO_2_.

### 5.4 The Role of Dopant and Surface Metal Species

The photodegradation of pollutants was observed on doped Nb_2_O_5_ and M/Nb_2_O_5_ catalysts under visible light irradiation (Table 1). The optical absorption ability on doped Nb_2_O_5_ catalysts was revealed by the experimental and theoretical analysis. For instance, an energy level sourced from N 2p orbitals was higher than that of the conduction band of O 2p states in pristine Nb_2_O_5_, leading to low bandgap energy of N–Nb_2_O_5_ (≈2.61 eV).^[^ ^19^ ^]^ In addition, the doping level formed by metal dopants was lower than that of the conduction band on pristine Nb_2_O_5_.^[^ ^85^, ^249^ ^]^ Meanwhile, the corresponding energy levels are still competent in the generation of O_2_^•−^ species for photodegradation. Furthermore, Nb_2_O_5_ catalysts modified by surface species can be active under visible light irradiation.^[^ ^171^ ^]^ Zhang's group reported the carbonate modified Nb_2_O_5_ for photodegradation of RhB under visible light irradiation.^[^ ^171^ ^]^ The *E*_g_ of C–Nb_2_O_5_ was increased to 3.06 eV after 500 °C calcination, suggesting the carbonaceous species for the enhanced visible‐light harvesting.^[^ ^171^ ^]^ Similar phenomena were also observed in N modified and C, N comodified Nb_2_O_5_.^[^ ^117^, ^172^, ^173^ ^]^ This can be ascribed to the transfer of electrons from surface NO*_x_* and CO*_x_* species to Nb_2_O_5_ under irradiation, which is analogical of dye‐sensitized photocatalysis.^[^ ^173^ ^]^ For M/Nb_2_O_5_ catalysts, the surface plasmon resonance (SPR) effect of metal species (e.g., Ag, Au, and Cu) is conducive to enhance the response to the visible light irradiation, ascribed to the match between the frequency of the incident light photons and the frequency of surface electrons on metal species.^[^ ^113^, ^206^, ^225^ ^]^

The catalysts structure and properties are also changed by the introduction of dopants on Nb_2_O_5_‐based photocatalysts, including the SSA, concentration of oxygen vacancies, and acidity. For instance, the SSA of doped Nb_2_O_5_ catalysts was higher than that of pristine counterpart.^[^ ^82^, ^85^ ^]^ These phenomena may be ascribed to the lattice distortion and inhibition of crystal growth by heteroatoms.^[^ ^82^, ^85^ ^]^ Besides, the concentration of oxygen vacancies can be increased when Nb_2_O_5_ catalysts were doped with N, Zr, Y, Zn, or Mo species.^[^ ^77^, ^90^, ^184^ ^]^ These phenomena were also observed on other metal oxides, like Cu–CeO_2_.^[^ ^250^ ^]^ As a result, the photocurrent density of N–Nb_2_O_5_ was higher than that of the pristine one.^[^ ^12^, ^19^ ^]^ The recombination efficiency of charge carriers is also changed with the concentration of oxygen vacancies, implying an optimal concentration of oxygen vacancies on the Nb_2_O_5_‐based photocatalysts in photocatalysis. In the future, machine learning is a promising tool to predict the structure and performance of catalysts.^[^ ^251^ ^]^

In addition, the acidity of Nb_2_O_5_‐based photocatalysts is also influenced by the dopant and surface metal species. Wolski's group reported ≈40% selectivity of dimethoxymethane (DMM) from methanol on Au/Nb_2_O_5_ while <5% selectivity of DMM on Nb_2_O_5_ was observed under UV light irradiation.^[^ ^225^ ^]^ The DMM is produced by the condensation reaction between the formaldehyde from the oxidation of methanol and adsorbed methanol molecules, implying the significantly decreased acidity of Nb_2_O_5_ after the introduction of the Au species.^[^ ^225^ ^]^ As revealed by FT‐IR results, the number of LASs decreased faster than that of BASs.^[^ ^225^ ^]^ Meanwhile, the numbers of BASs and LASs were distinct by different preparation approaches.^[^ ^225^ ^]^ These may be ascribed to the formation of Nb^4+^ species from the reduction of Nb^5+^ in NbO_4_ units by hydrogen spillover in the reducing atmosphere.^[^ ^14^, ^252^ ^]^ Besides, the BAS is possibly neutralized by basic additives in the deposition–precipitation approach.^[^ ^8c^ ^]^ Occasionally the adsorption of the substrate is enhanced by the dopant on Nb_2_O_5_‐based photocatalysts. The adsorption of methylene blue molecules on Zr–Nb_2_O_5_ is considerably stronger than that over the pristine counterpart.^[^ ^77^ ^]^ Similarly, the intact interaction between methyl violet molecules and Mo–Nb_2_O_5_ cluster was speculated, as revealed by the results of surface‐enhanced Raman scattering (SERS) and first‐principles calculation.^[^ ^90^ ^]^ Furthermore, CO was obtained with a selectivity of 99.5% from the hydrogenation of gaseous CO_2_ using the small Pd nanocrystals supported on Nb_2_O_5_.^[^ ^253^ ^]^ Density functional theory (DFT) calculations suggested that the Pd(111) facets dominated on the larger nanoparticles were the most favorable sites for methanation of CO_2_.^[^ ^253^ ^]^

### 5.5 The Role of Formed Heterojunctions

Previously, Zheng's group synthesized an amorphous layer on TT‐Nb_2_O_5_ microfibers (HN‐500).^[^ ^254^ ^]^ As revealed by the results of VB XPS spectra, the edges of the maximum energy for and TT‐Nb_2_O_5_ were identical with that of the amorphous counterpart.^[^ ^254^ ^]^ Meanwhile, the bandgap of amorphous‐phase is ≈0.2 eV higher than that of TT‐Nb_2_O_5_, indicating the formation of heterojunction on HN‐500.^[^ ^254^ ^]^ The electronic structure of heterojunction has an advantage in the separation of charge carriers Besides, short‐range ordered Nb_2_O_5_ can be dispersed on the surface of amorphous structure, leading to the formation of the interface between amorphous and ordered Nb_2_O_5_.^[^ ^166^ ^]^ This may be conducive to the desorption of desired products, due to the distinction in acid strength on different phases.^[^ ^14a^ ^]^

In addition, the spatial distribution of charge carriers can be observed in other Nb_2_O_5_‐based photocatalysts.^[^ ^255^ ^]^ The type II heterojunctions were obtained by modification of Nb_2_O_5_ with other composites, such as TiO_2_, WO_3_, ZnO, CdS, C_3_N_4_, Ag_3_PO_4_, SrNb_2_O_6_, BiWO_4_, BiNb_5_O_14_, and so on.^[^ ^99^, ^100^, ^101^, ^116^, ^160^, ^162^, ^181^, ^191^, ^205^ ^]^ For instance, electrons transfer from the CB of Nb_2_O_5_ to the CB of BiWO_4_ while the holes migrated from the VB of BiWO_4_ to the VB of Nb_2_O_5_ under UV light irradiation, leading to limited redox potentials of Nb_2_O_5_/BiWO_4_.^[^ ^191^ ^]^ When Nb_2_O_5_ was deposited on the ZnIn_2_S_4_, a Z‐scheme heterojunction was observed.^[^ ^217^ ^]^ Partial photogenerated holes and electrons were still present on the VB of Nb_2_O_5_ and CB of ZnIn_2_S_4_, respectively. This electronic structure can maintain the oxidizing potential of Nb_2_O_5_ and reducing the capacity of ZnIn_2_S_4_, respectively.^[^ ^217^ ^]^ In Z‐scheme heterojunction, partial electrons are transferred from the CB of Nb_2_O_5_ to the VB of ZnIn_2_S_4_, which is attributed to photocatalytic performance.^[^ ^217^ ^]^ Similar heterojunction structures were also observed on Er–Y_3_Al_5_O_12_@Nb_2_O_5_/Pt/In_2_O_3_ composite catalyst.^[^ ^214^ ^]^

Especially, the interface is present in composited Nb_2_O_5_ catalysts.^[^ ^227^ ^]^ Previously, Cu_2_O/Nb_2_O_5_ exhibited higher activity than that of the pristine counterpart in the photooxidation of alcohol.^[^ ^248^, ^256^ ^]^ As revealed by FT‐IR results, the adsorption of cyclohexanone over Cu (I) was weaker than that over Nb^5+^ species in the photooxidation of cyclohexanol. ^[^ ^256a^ ^]^ Meanwhile, the oxidative dehydrogenation of alcohols was realized on Nb^5+^ species, indicating the accelerated catalytic recycle on Nb—O—Cu (I) interface.^[^ ^248^ ^]^ To this end, a series of Nb_2_O_5_‐based catalysts have been widely reported in the photocatalytic process. However, few studies focus on the role of the catalyst interface in photocatalysis.

## 6 Summary and Outlook

This review summarized recent advances in the synthesis and application of Nb_2_O_5_‐based photocatalysts. Especially, the reaction pathways in the reduction of CO_2_, oxidation of amines, alcohols, and hydrocarbons are related to the acidity, generated oxygen species, and functional groups on Nb_2_O_5_‐based photocatalysts under light irradiation. The understanding greatly relies on the studies in the local structure of Nb_2_O_5_, which is still attractive for researchers, especially in the field of photocatalysis. The universal characterization techniques and photoelectronic properties are the cornerstones to reveal the active sites of Nb_2_O_5_‐based photocatalysts.

In the future, Nb_2_O_5_‐based photocatalysts are still the potential candidates for the conversion of waste plastics and biomass that are abundant carbon resources. The activations of sp^3^ C—H bonds and C—C bonds are feasible over Nb_2_O_5_ that exhibits suitable photoredox potentials under light irradiation. Although the yield and selectivity of desired products are limited in the conversion of hydrocarbon and biomass, the reactivity of Nb_2_O_5_ can be further improved by the design of catalyst structure and components. Besides, the local structure and corresponding acidity strength can be in situ controlled on Nb_2_O_5_ under light irradiation. As a result, it is possible to realize the hydrolysis reaction, dehydration reaction, and hydrodeoxygenation reaction under mild conditions. Using the blueprint of photocatalysis, we can improve the conversion of waste plastics and renewable biomass.

Despite the great potential of Nb_2_O_5_‐based photocatalysts, the exposed challenges and issues should be considered. The roles of NbO_4_ and NbO_6_ units are unclear in photocatalysis, due to the lack of spatiotemporal characterization technique. Apart from that, the studies are insufficient in the interface of Nb–O–metal, which can afford the profound understanding of the adsorption–desorption process and active sites in photocatalysis. Meanwhile, the development of the reactor is conducive to the practical application of the photocatalytic process.^[^ ^257^ ^]^ Studies in these aspects are necessary for the rational design of Nb_2_O_5_‐based photocatalysts. Moreover, novel processes and concepts are urgently required for the large‐scale production of Nb_2_O_5_‐based photocatalysts.

## Conflict of Interest

The authors declare no conflict of interest.
